# From “skype on wheels” to embodied telepresence: a holistic approach to improving the user experience of telepresence robots

**DOI:** 10.1007/s10055-025-01222-0

**Published:** 2025-09-25

**Authors:** Ivan A. Aguilar, Markku Suomalainen, Steven M. LaValle, Timo Ojala, Bernhard E. Riecke

**Affiliations:** 1 Simon Fraser University, Surrey, British Columbia Canada; 2 VTT Technical Research Centre of Finland, Oulu, Finland; 3 University of Oulu, Oulu, Finland

**Keywords:** Virtual reality, Telepresence robot, User interface, Navigation, Spatial presence, Empirical user study

## Abstract

Telepresence robots offer the promise of remote presence, but user experience, usability, and performance challenges hinder widespread adoption. This study introduces a novel and low-cost user interface for telepresence robots that integrates insights from virtual reality (VR) and robotics to address these limitations. The novel setup was designed holistically, considering several different factors: an inclined rotating chair for embodied rotation, a joystick for precise translation, dual displays for enhanced spatial awareness, and an immersive setup with controlled lighting and audio. A user study (N = 42) with a simulated robot in a virtual environment compared this novel setup with a standard setup, that mimicked the typical user interface of commercial telepresence robots. Results showed that this novel setup significantly improved the user experience, particularly increasing presence, enjoyment, and engagement. This novel setup also improved task performance over time, reducing obstacle collisions and distance traveled. These findings highlight the potential for combining and incorporating insights from VR and robotics to design more effective and user-friendly interfaces for telepresence robots, paving the way for increased adoption.

**Supplementary Information:**

The online version contains supplementary material available at 10.1007/s10055-025-01222-0.

## Introduction

**Fig. 1:**
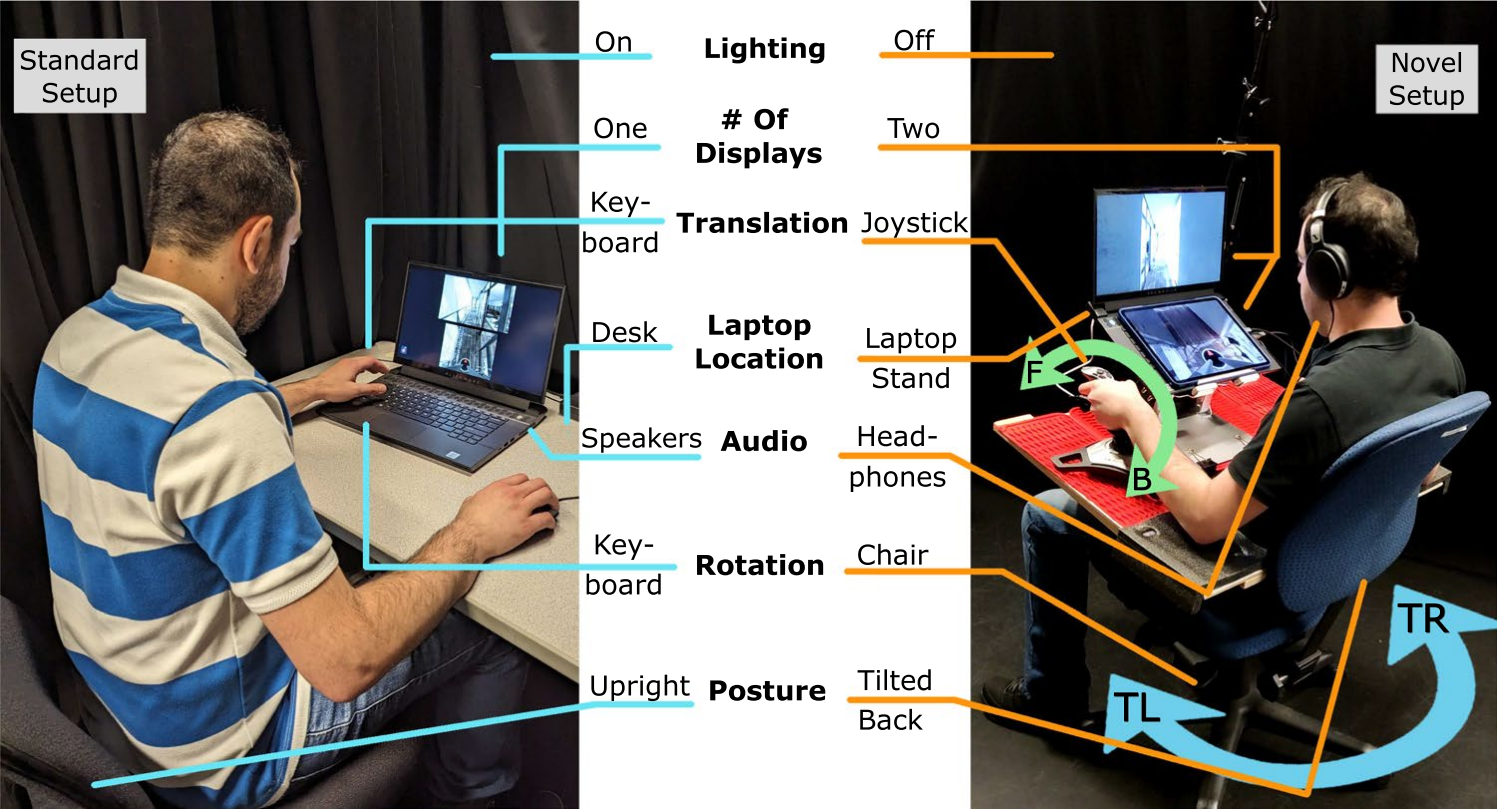
Setups used in the study and their respective factors. The Standard setup (left), has lights on, one display, keyboard for translation input (W/S keys), laptop is placed on a desk, speakers used for audio, keyboard for rotation input (A/D keys), and chair is at an upright posture. The Novel setup (right), has lights off, two displays, joystick for translation input (push forward and pull backward), laptop is placed on a laptop stand, headphones for audio, chair/physical rotation for rotation input (turn left/right), and chair is at a tilted back posture. For the Novel setup, the joystick and laptop stand are placed on a wooden platform attached to the chair’s armrest to allow everything to rotate with the chair

Teleoperated robots allow users to view, navigate through, and communicate with those in a remote environment (Desai et al. 2011; Almeida et al. 2022), offering an improved sense of presence and agency over traditional forms of long-distance communication (Rae et al. 2014; Yang et al. 2018; Kristoffersson et al. 2013). Telepresence robots find applications in diverse fields. In business, employees can remotely work and attend meetings (Beno 2018; Lee and Takayama 2011); in security and safety, safety personnel can remotely patrol areas, navigate dangerous environments, or conduct search and rescue missions (Schultz et al. 1991; Henkel et al. 2016); in education, students can remotely attend classes and participate in group projects (Newhart 2014; Yousif 2021; Velinov et al. 2021), and attend conferences (Neustaedter et al. 2016, 2018); in health, health professionals can remotely monitor and consult with patients (Vespa et al. 2007; Seethalakshmi et al. 2021; Kristoffersson et al. 2011); in personal home use, users can check on their home and family (Yang and Neustaedter 2018; Hung et al. 2022); and it is a device that enables people with accessibility challenges to navigate and perform tasks (Neustaedter et al. 2016; Zhang and Hansen 2022; Tsui et al. 2015); among other uses (see reviews (Tsui et al. 2011; Murphy et al. 2020)).

Despite their potential, telepresence robots have not yet achieved widespread commercial adoption. One issue users have with these robots is encompassed by the informal pejorative comment that they feel like "Skype on wheels", as users have reported difficulties and unnatural interactions with them, negatively impacting their experience and performance of tasks in a remote environment compared to physically being there (Stoll et al. 2018). This may be due in part to a lack of compelling presence (a sense of being and acting realistically in a remote environment), which contributes to users behaving in unnatural ways (Slater 2009; Slater et al. 2009). This may also be due to usability issues of the user interface, demanding a high cognitive load to control navigation and lacks spatial awareness and peripheral vision (Neustaedter et al. 2016; Hauser et al. 2025), keeping users mainly focused on safely operating and maneuvering the robot rather than their primary task (Cohen et al. 2011; Hauser et al. 2025), for instance engaging in conversations with others (Lee and Takayama 2011; Yang et al. 2018), negatively affecting performance (Rae et al. 2014). Addressing these presence and navigation issues is paramount to improving the user experience, performance, and, consequently, the widespread adoption of telepresence robots.

A potential way to investigate these issues is to observe how they are addressed in a domain with similar challenges. Virtual reality (VR) is a field that uses devices to allow users to visualize, navigate in, and interact with a three-dimensional fully virtual (computer-generated) environment (Burdea and Coiffet 2003; Milgram et al. 1995). The field of VR shares certain similarities with telepresence robots that warrant consideration. For instance, how users: visualize their virtual/remote environment (displays used), control their view of it (virtual/robot camera control), navigate through it (input devices), and the challenges users face when using them (user experience, usability, and performance). Given these similarities, we aim in this paper to leverage the knowledge and concepts from VR studies to design and evaluate navigation methods to improve the overall experience users have when using telepresence robots.

One VR concept is spatial presence, the user’s consistent sense of being in a virtual environment (VE) (Lee 2004; Slater 2018) while intuitively having a spatial awareness of their virtual surroundings (understanding where they are and where/how far virtual objects are from them) (Riecke and Heyde 2002). This awareness is important because users often get lost while navigating in VEs (Chance et al. 1998; Ruddle and Jones 2001) and they misperceive distances compared to the real world (Creem-Regehr et al. 2003; Witmer and Singer 1998; Thompson et al. 2004; Riecke et al. 2005). The input method users use to navigate across a VE is important (Boletsis 2017; Cherni et al. 2020; Martinez et al. 2022), it needs to have good usability and provide users with a good user experience (Kim et al. 2020; Kazemi and Lee 2023) while optimizing task performance (Bowman et al. 2002) and minimizing cybersickness (also known as simulator or motion sickness) (Keshavarz and Golding 2022; Keshavarz et al. 2015; Khundam 2021; Berger and Wolf 2018). Another related concept is sensory immersion, where a device can overpower (Ermi and Mäyrä 2005) and obstruct real-world distractions and sensory cues (Zheng et al. 1998) by submerging the user’s multisensory perceptions in the environment presented to them (Biocca and Delaney 1995). Immersion can also enhance users’ attention (Cho et al. 2002), sense of presence (Draper et al. 1998), focus (Brown and Cairns 2004), and performance on cognitive tasks like spatial understanding, spatial memory (Dinh et al. 1999; Schuchardt and Bowman 2007; Tan et al. 2003), recall, and mental map development (Sowndararajan et al. 2008).

To explore how the experience of controlling a telepresence robot could be improved, we leveraged knowledge from both VR (human-computer interaction (Dix 2009)) and robotics (human-robot interaction (Thrun 2004)) to design a new user interface (hereafter referred to as "Novel setup"). This interface specifically focuses on the information transferred between the robot operator (input) and the robot’s system (output). We then conducted a user study to investigate how this Novel setup compares to a control/baseline user interface typical of telepresence robots (hereafter referred to as "Standard setup") (Rae and Neustaedter 2017; Björnfot 2021; Yang et al. 2018), across various aspects, including presence, user experience, usability, and performance. To better control the environment and avoid confounding variables, this comparison between interfaces was conducted with a simulated robot traversing a VE.

**Contributions.** We contribute the following:

1. **A holistic and accessible interface paradigm** that integrates principles of embodiment and immersion to fundamentally enhance the telepresence experience, all without requiring hardware modifications to the robot itself.
2. **Rigorous empirical evidence** from a user study (N = 42) demonstrating that this paradigm significantly improves presence, enjoyment, engagement, and task performance over time when compared to a conventional interface that mimics typical commercial systems.
3. **A nuanced analysis of the critical trade-offs** inherent in highly immersive systems, offering insights into the challenges of motion sickness, physical demand, and other aspects that must be considered in the design of future telepresence interfaces.

## Related works

In this section, we present how other studies have investigated modifications to robot user interfaces and key constructs and measures used to evaluate telepresence robot setups: presence, user experience, usability, and performance.

### Presence

The sense of presence is central to telepresence systems, as it influences how naturally users interact with the remote environment (Kristoffersson et al. 2013; Minsky 1980; Batmaz et al. 2020). Research on VEs has shown that non-immersive setups result in lower levels of presence compared to immersive setups (Slater 2018), which may cause users to behave in unnatural ways (Slater 2009). This has implications for telepresence robots, where the standard non-immersive setup may hinder the user’s sense of presence and lead to users having difficulty performing tasks (Stoll et al. 2018). Although the use of a VR head-mounted display (HMD) as a setup’s display may be an obvious factor to enhance presence, other factors can influence it, such as the input device, the number of camera views (Adamides et al. 2017), and the user’s posture (Kim et al. 2019, 2020).

A strong sense of presence generally improves performance, as users who feel more strongly in the environment can better understand and engage in tasks (Grassini et al. 2020; Maneuvrier et al. 2020; Draper et al. 1998; Nash et al. 2000). However, the attention and cognitive demand required both from the virtual (i.e., spatial understanding and obstacle avoidance) and the real world environment (i.e., input device manipulation) can affect both presence and performance in different ways, meaning that the relationship between performance and presence depends on context (Maneuvrier et al. 2020), task, attention, and cognitive demand (Draper et al. 1998; Nash et al. 2000). Beyond presence, it is also important to consider the user experience, usability, and performance implications of different telepresence robot setups.

### User experience, usability, and performance

User experience, usability, and performance are significantly affected by the input device used for navigation (Slater et al. 2009; Van Erp et al. 2006; Randelli et al. 2011; Doisy et al. 2017; Nash et al. 2000). Studies have found that the input device used can significantly improve task completion time, reduce the number of collisions (especially in confined spaces), and that not only the naturalness and intuitiveness of a device should be considered, but also its sensitivity, cognitive demand (Randelli et al. 2011), and affordances (Riecke and Zielasko 2020). The amount of trials/time spent using a setup is also important, as it can give users enough opportunity to both learn how to use and improve their performance (Doisy et al. 2017).

### Translation methods

Studies have investigated multiple methods for maneuvering robots in remote environments that exceed the user’s physical space. Using keyboard presses to control robot navigation is common not only in academic research (Bazzano et al. 2019; Wang et al. 2013; Leeb et al. 2015; Rae et al. 2014; Yang et al. 2018; Bazzano et al. 2017; Guo and Sharlin 2008; Kadous et al. 2006) but also in commercial telepresence setups (Björnfot 2021). A study showed that setups using a keyboard allowed for faster task completion, showed greater perceived usability, and required less workload when compared to setups using a gamepad as input (Adamides et al. 2017).

Another method used to navigate robots is redirected walking, which allows users to walk in curved paths which are perceived as straight-lines, but it requires large rooms and tracking equipment (Zhang et al. 2018; Li et al. 2022). Omnidirectional treadmills have also been used to locomote robots, as they allow for continuous walking in any direction, but impose unnatural motions (e.g., slipping and sliding walking gaits) from users, causing delays and mismatches between user input and robot movement due to bio-mechanical differences between humans and robots (Spada et al. 2019; Elobaid et al. 2019). These methods are also costly, limiting their practicality; see (Nilsson et al. 2018) for a comprehensive review of walking locomotion methods.

The use of Brain-computer interfaces (BCIs) (Bell et al. 2008; Leeb et al. 2015; Escolano et al. 2011) and gestures (Jiang et al. 2012) have also been explored, but they are more cognitively and physically demanding, respectively, and do not perform as well as other methods, such as a gamepad controller’s joysticks (Doisy et al. 2017).

### Rotation methods

We humans rotate our head to change our viewpoint and better understand our real-world surroundings; similarly, it is more intuitive and immersive for users to rotate their head to see more of a displayed environment rather than just look in a fixed direction. Stationary displays don’t follow head rotations, thus breaking immersion if users rotate their head (Slater 2009). VR HMDs address this problem by having the display accompany users as they turn, providing robot operators with an easier, more natural, and more immersive experience (Pittman and LaViola Jr 2014) rather than relying on virtual rotation methods that use input from devices such as gamepads, joysticks, or keyboards to rotate the robot (Doisy et al. 2017; Björnfot 2021). Despite these benefits, HMDs have drawbacks, including performance, user experience, and, in particular, motion sickness (Adamides et al. 2017; Chen et al. 2013; Ha et al. 2015; Koenemann et al. 2014; Suomalainen et al. 2021), especially as the time spent in VR (exposure duration) increases (Rouhani et al. 2024; Saredakis et al. 2020; Kennedy et al. 2000), which is a concern when telepresence robots are often used for long sessions (i.e., over 20 min) (Jones et al. 2020; Yang et al. 2018; Shi et al. 2019; Cash and Prescott 2019). When used to remotely control telepresence robots, HMDs are limited in that they cannot follow the user’s head pose. This is because the robot’s camera is not omnidirectional, it is fixed to its base, and does not typically move or rotate with the user (Luo et al. 2023). This can cause nausea and discomfort due to discrepancies between what the user expects to see when they move their head and what they actually see (e.g. the user moves their head to the left, but the view does not change because the robot’s camera did not move with them).

One method of controlling a robot’s rotation (also known as forward direction or heading) without the use of virtual rotations is to allow the user to rotate their chair rather than using a joystick (Van Erp et al. 2006). This method was preferred by users as they were more careful and moved the robot more smoothly, reached maximum rotation and translation speeds less often, and often rotated without translating at the same time. There are also approaches that allow users to rotate their view independently of their navigation direction by tracking the user’s head to rotate the camera view (Doisy et al. 2017; Hughes et al. 2003; Pathak et al. 2016; Suomalainen et al. 2022) while the direction of movement is controlled by another input, such as a gamepad (Doisy et al. 2017), hand gestures (Doisy et al. 2017), the user’s head direction (Zielasko et al. 2020; Hashemian et al. 2024, 2023; Nguyen-Vo et al. 2019), or their hip/torso direction (Zielasko et al. 2020). While this allows users to look and perform a task in one direction while moving in another, controlling robots in this way requires tracking devices to track users and modifying the robot’s hardware, either using a 360 camera and/or decoupling the camera from its base so that it can rotate independently (212).

See these guidelines and reviews for further detail on designing teleoperated robots with a focus on locomotion methods and inputs (Gifford 2006), user experience and usability (Desai et al. 2011; Adamides et al. 2014; Youssef et al. 2023), task performance (Bostelman et al. 2016), and presence, usability, and performance in VEs (Nash et al. 2000; Chandra et al. 2019; Wilkinson et al. 2021).

## Designing the novel telepresence system

### Design pillars

We grounded our setup on six "design pillars" - a design concept that defines elements or emotions that the experience is trying to achieve (Pears 2017). These design pillars were used to brainstorm high-level goals and guidelines to focus development (Despain 2012). We focused on how to support the telepresence robot user’s sense of presence, user experience, usability, and performance. Our group consisted of experts in the fields of VR and robotics, and we drew ideas from the literature in these fields and from our personal experiences. Through our discussions, we agreed on these design pillars that the setup should provide to users:

**(1) Agency in the Robot’s Control**: users should feel in full control of the robot;

**(2) Intuitive and Precise Control**: users should perceive their control of the robot to be intuitive and precise;

**(3) Spatial Awareness of the Remote Environment**: users should easily understand what is presented to them so they can effectively search and navigate the remote environment;

**(4) Immersive Setting**: users should feel immersed and be able to easily focus on the remote environment;

**(5) Comfort**: users should feel comfortable when controlling the robot.

**(6) Modular, Affordable, and Accessible**: users should be able to affordably and easily modify their setup to achieve the desired setup.

Next, we describe each of these design pillars, drawing on relevant literature to support and explain our rationale for incorporating them into our setup.

### Agency in the robot’s control

We provided users with full manual control of the telepresence robot (also known as pure teleoperation) because some users prefer manual control to having the robot’s system influence their navigation (i.e., using shared or autonomous control for maneuvering) because of the sense of agency it provides (Batmaz et al. 2020). Furthermore, manual control is a common control method for teleoperated robots (Rae et al. 2014; Yang et al. 2018; Guo and Sharlin 2008; Björnfot 2021), usually an available option in case of system failure or problems (i.e. the robot is unable to perform a task autonomously, the user cannot point to or reach a desired location because the environment is too cluttered or narrow, etc.) (Chen et al. 2013; Armbrust et al. 2010; Eck et al. 2008), and is expected to be preferred by users for short-range motions.

Although manual control in a final telepresence robot product would include various types of guarded motions and collision detectors, we did not include them in our robot’s system because they could confound the analysis of how users use the interfaces to maneuver and avoid obstacles without the help of the robot’s system.

### Intuitive and precise control

#### Translation input: joystick

We decided to use a joystick as the translation input device for the Novel setup for the following reasons: Joystick input is commonly used for robot control (Pang et al. 2014; Hainsworth 2001; Guo and Sharlin 2008; Chestnutt et al. 2006; Baker et al. 2020; Doisy et al. 2017; Stotko et al. 2019); it is portable and provides a large range of motion, reducing the user’s need for trained fine-motor skills compared to a gamepad’s thumbstick; it is stationary; requires only one hand to operate, compared to gamepads which require both hands to hold and operate (Björnfot 2021); and provides improved force-feedback, which is preferred by users (Kechavarzi et al. 2012). Joysticks are also commonly used in the control of powered wheelchairs (Fehr et al. 2000; Dolan and Henderson 2017; Cooper et al. 2002; Sorrento et al. 2011; Koyama et al. 2023; Leaman and La 2017), which have similar movement limitations and control tasks. Furthermore, joysticks were recommended by participants as an input device that could improve the control of telepresence robots (Björnfot 2021).

#### Rotation input: physical rotation

Physical rotation (or real, embodied, self, body-based, or turn-in-place rotation) is a commonly used rotation method used with VR HMDs as it allow users to rotate their viewpoint of a displayed environment by physically turning in their real world (Schubert et al. 1999; Hollerbach 2002). This rotation method was preferred over other rotation methods (Van Erp et al. 2006) and when compared to virtual rotation improves task performance, such as search efficiency and task completion time (Riecke et al. 2010), spatial cognition tasks, like spatial updating and navigational search (Ruddle and Lessels 2006, 2009; Klatzky et al. 1998; Presson and Montello 1994; Rieser and Rider 1991; Chance et al. 1998; Riecke et al. 2010), with performance levels almost approaching those of actual walking (Riecke et al. 2010). See (Ruddle 2013) for a thorough review on this topic.

To circumvent the problems associated with VR HMDs and to investigate the limits of using embodiment and immersion techniques for telepresence without the use of an HMD, we decided not to use an HMD as the display in our setup. Instead, we sought to preserve the benefits of physical rotation by tracking the user’s chair rotation to rotate the robot’s viewpoint. To have the monitor and other devices accompany the user’s chair rotation, we mounted them on a platform attached to the chair’s armrests, as shown in Figure 1.

### Spatial awareness of the remote environment

The quality and properties of the display are pivotal for telepresence robots, as users rely heavily on video feed from the robot’s camera(s) for spatial awareness so they can easily understand and effectively navigate the remote environment (Rae et al. 2015). One important aspect is display size, where larger displays positively affect the user’s ability to recall what was presented to them (Detenber and Reeves 1996), as well as their enjoyment and overall positive user experience (Lombard and Ditton 1997; Grabe et al. 1999). Another key aspect is field-of-view (FoV), the angular extent a user/sensor can observe. The camera on many robots is fixed to the base, which restricts its mobility. This, when coupled with a restricted FoV, can reduce spatial awareness and impair the user’s perception of the robot’s surroundings (Hauser et al. 2025; Bazzano et al. 2017; Neustaedter et al. 2016; Yanco and Drury 2004). This limited FoV can lead to ’tunnel vision’ and detachment from the remote environment (Vaughan et al. 2016), making even simple teleoperated tasks challenging (Chen et al. 2007; Yanco and Drury 2004).

A potential solution is to increase the user’s FoV as larger FoVs require less attention and cognitive demand from users (Bazzano et al. 2019; Marsh et al. 2013), improve task completion times (Bazzano et al. 2019; Nagahara et al. 2003; Wells et al. 1988; Johnson et al. 2015), improve target detection and identification performance (Ragan et al. 2015), increase the user’s sense of presence (Lin et al. 2002), reduce the number of collisions (Nagahara et al. 2003; Johnson et al. 2015), require less navigation commands (Bazzano et al. 2019), and enhance distance judgment (Masnadi et al. 2022). However, larger FoVs can also increase the risk of motion sickness, which may negatively impact user enjoyment (Lin et al. 2002).

Multiple techniques exist to extend a robot’s FoV. For instance, the camera can be rotated (Nielsen et al. 2005; Bazzano et al. 2017; Vaughan et al. 2016; Hauser et al. 2025) and moved (Schwarz and Behnke 2021) based on user input (Doisy et al. 2017) or by tracking and matching the user’s head motions (Doisy et al. 2017; Hauser et al. 2025). However, these techniques would require additional cognitive resources from the user (Vaughan et al. 2016) and may induce motion sickness if translations/rotations are inconsistent, or if there is a noticeable delay in relation to the user’s motion (Hauser et al. 2025).

Another way to enhance spatial awareness, particularly depth perception (useful for estimating distance and avoiding obstacles), is to use dual cameras to provide users with stereoscopy and motion parallax instead of the usual single, monoscopic camera view (Livatino et al. 2009; Hauser et al. 2025). Depth sensors, such as LiDAR (light detection and ranging) or RGB-D (color and depth), can be used to create a map of the environment showing more than the main camera view (Stotko et al. 2019; Chen et al. 2022). Alternatively, 360-degree/omnidirectional cameras can be used to provide a large FoV (Suomalainen et al. 2022; Heshmat et al. 2018; Wibowo et al. 2021; Chandan et al. 2021). However, implementing these solutions would require hardware changes to typical telepresence robots.

Using multiple camera views may also improve spatial understanding and navigation, as improvements in obstacle avoidance, task completion times (Adamides et al. 2017), spatial awareness and sense of presence have been demonstrated (Nakano et al. 2021). However, the position of the cameras is important, as a third-person, bird’s-eye, or overhead camera views can be used to show the user and their surroundings (Adamides et al. 2017; Hauser et al. 2025), but this decreases the user’s sense of presence and immersion (Rouse 1999; Denisova and Cairns 2015). A solution could be to use two cameras, a forward-facing camera, pointed forward to show where the robot is going, and a down-facing camera, pointed downwards to show the robot’s immediate surroundings, which helps to avoid obstacles and is present in some telepresence robots (Herring 2013; Neustaedter et al. 2016; Heshmat et al. 2018; Sobrepera et al. 2021).

Given these considerations of display size, FoV size, and number of camera views, and with users potentially having portable displays like tablets and phones readily available to them, and with laptops starting to have multiple displays (Lenovo 2024; Asus 2024), we designed our setup to include an additional display. This display was positioned below the primary screen and at a downward angle, each displaying a unique camera view. This display arrangement increases the overall display size and FoV, potentially improving spatial awareness while providing the affordance of looking forward and downward to view different aspects of the remote environment, similarly to how we view things in the real-world environment.

### Immersive setting

Research has shown that decreasing light levels and blocking out distractions decreases awareness of real-world surroundings (Zheng et al. 1998), allowing users to focus on what is being presented, which enhances immersion (Nordin et al. 2014). Specifically, having the room lights off while increasing the content’s volume decreases distractions from the user’s real-world surroundings and helps induce and not break presence (Brown and Cairns 2004; Kitson et al. 2020).

To enhance immersion and reduce external distractions in the Novel setup, we opted to have the lights off and use noise-canceling headphones - a common setup used in VR studies (Kitson et al. 2020; Freiberg et al. 2013; Johnson and Coxon 2016; Kern et al. 2016; Skalski et al. 2011).

### Comfort

Comfort is affected by the user’s posture, where sitting in a back-tilted position reduces physical strain (Bendix et al. 1985) and is more comfortable than an upright posture (Haynes and Williams 2008). The use of armrests provides support for the user’s upper arms, especially in tilted postures (Haynes and Williams 2008). Additionally, placing a display at eye-level can further enhance comfort (Kothiyal and Bjørnerem 2009; Imamov et al. 2020). Aside from the obvious influence on comfort, posture can also affect the user’s performance (Imamov et al. 2020), their sense of presence (Kim et al. 2019, 2020), and cognitive performance (Isip 2014; Patston et al. 2017; Schulman and Shontz 1971), specifically attention (Barra et al. 2015; Caldwell et al. 2003; Rosenbaum et al. 2017), working memory, and executive function (Mehta et al. 2016).

Because of these benefits of a comfortable posture, we chose to have the user’s chair at a back-tilted angle and a laptop placed on a laptop stand to be at eye-level, fixed to the chair’s platform (discussed in section 3.3.2).

### Modular, affordable, and accessible

A constraint we imposed on our design was to not require any modifications to the robot itself (its hardware). This constraint reduces cost and development time and allows this setup to potentially be used for different types of robots with similar capabilities (i.e., wheeled mobile robots that can rotate in place). The only modifications required are on the user side, and they must be affordable and easy to accomplish, requiring only simple and inexpensive changes to a typical user interface, using equipment that users may already have or that could be easily acquired. The design must also be modular, allowing for rapid prototype iterations and potentially allowing users to pick and choose factors to include in their setup.

Through iterative prototyping and conducting pilot studies to investigate prototypes, the final version of our proposed setup had the following factors: a joystick to control the robot’s translation and physical rotation, via chair rotation detected using an inertial measurement unit (IMU) attached to the back of the laptop stand, to control rotation. The room lights were off, a laptop was on a laptop stand, the chair was in a back-tilted posture, and audio was provided via noise-canceling headphones. Two camera views were used, with a forward-facing camera view displayed on the laptop monitor and a down-facing view displayed on a tablet (secondary display) placed on the laptop’s keyboard. A custom cabling system was used above the setup to manage power, ensure consistent frame rates, and allow users to rotate without tangling cables. Though the hardware and techniques used are not new per se, the novel aspect is their integration and configuration in this setup.

## Methods

### Proposed design evaluation

To explore the effects of these design choices and users’ experience with using them to operate telepresence robots, we conducted a study comparing our Novel setup with a Standard setup across multiple measures, including presence, user experience, usability, and performance. This Standard setup mimicked the typical office and home setup/setting used to control telepresence robots: keyboard input to control both translation (W/S keys) and rotation (A/D keys) (Bazzano et al. 2019; Wang et al. 2013; Leeb et al. 2015; Rae et al. 2014; Yang et al. 2018; Bazzano et al. 2017; Guo and Sharlin 2008; Kadous et al. 2006), room lights on (Batmaz et al. 2020; Ishiguro et al. 2020; Koenemann et al. 2014; Rae et al. 2014; Neustaedter et al. 2016; Lee and Takayama 2011; Jones et al. 2020), laptop placed on a desk, upright chair posture, and audio is delivered through the laptop’s speakers, two camera views (vertically stacked) presented on a single display (Neustaedter et al. 2016; Heshmat et al. 2018). See Figure 1 to view both setups and their respective factors.

### Research questions

Based on the issues users have with operating telepresence robots, we sought to answer the following overarching research question (RQ) with this study: **How do the setup factors related to embodiment (e.g., physical rotation, joystick for translation, screen placement, posture) and immersion (e.g., number of displays, audio, lighting), over time, contribute to the sense of presence, user experience, usability, and performance?**

### Experimental design

The study had a $2\times 3$ within-subjects repeated measures design. There were two conditions, counterbalanced across participants, in which participants first used either the Standard or the Novel setup to complete tasks, repeated three times for each setup for a total of six trials.

### Dependent variables

To evaluate our setups in a wide range of aspects, we selected a total of 47 dependent variables. They consisted of 34 subjective/introspective (self-reported) variables to measure presence (five variables), user experience (seven variables), usability (twenty variables), and setup factor importance (two variables), and thirteen objective (behavioral) variables to measure performance (seven raw and six processed). These measures are detailed below, but for a more information on how they were recorded and/or calculated and what questionnaire or study they are based on see Section 1 in Supplementary material 1.

#### Presence

We asked participants four questions on: how aware they were of their real environment, which setup they felt more present in the simulated environment with, and had them answer the IPQ to calculate their presence and spatial presence scores. For vection, we asked participants how much vection they felt.

#### User experience

We asked participants seven questions on: tiredness - how tired they felt, relaxed muscles - how relaxed their muscles were, comfort - how comfortable it was to use the setup, engagement - how involved and engaged they felt, enjoyment - if they enjoyed using the setup, excitement - how exciting it was to use the setup, motion sickness - how motion sick they felt (using the total SSQ and its subscores).

#### Usability

We asked participants twenty questions on using the setup: safety - how safe it was, regular use - if they would it regularly, long term use - if they would use it for long periods of time, complicated - how complicated the setup was, confusing to use - how confusing it was to use, precise control - if they had precise control of their movements, overall usability - if overall usability was high, movement speed - what they thought of the movement speed, easy obstacle avoidance - if it was easy avoid obstacles, task efficiency - how efficient it was to complete the task, task support - how supportive it was to complete the task, ease of learning - if it was easy to learn how to navigate, easy task concentration - if they could easily concentrate on the task, task load - how much task load the setup required of them (using the overall NASA-Task Load Index and sub-measures), and easier to control - which setup was easier to control the robot with.

The five remaining questions focused on how suitable setups may be in different use cases. We asked participants which setup they would prefer to use by having them imagine different situations. The questions were which setup they would prefer to use a telepresence robot to remotely: travel or sightseeing - exploring places they would like to visit, personal social situations - like meeting with friends and family, small professional gatherings - like a business visit in a different city, large professional gatherings - like conferences or events, task-focused - like the one in the study (navigating, finding things, avoiding obstacles).

#### Behavioral measures

We recorded seven raw behavioral measures during task completion. These included: **task completion time** (the total duration of the trial), the **number of collisions** with environmental objects, **collision time** (the cumulative duration the robot was in contact with an object), **distance traveled**, **accumulated rotations** (total rotation in the yaw axis), **average linear speed** (excluding time spent interacting with the graphical user interface), and **average rotation speed**.

To facilitate a more nuanced evaluation of task performance, we processed these raw behavioral measures into six efficiency indexes. Four of these indexes—for task completion time, number of collisions, distance traveled, and accumulated rotations—were defined as the ratio of the Standard setup’s value to the Novel setup’s value (Standard / Novel). Thus, a value greater than 1 signifies a performance advantage for the Novel setup, while a value of 1 indicates parity. To account for the explicit instruction to balance speed and precision (Sect. 4.6), we developed a composite performance efficiency index based on the geometric mean of the task time and collision data (see Section 2 in Supplementary material 1 for details). Furthermore, because these base measures all contribute to a robot’s power draw, we derived an energy efficiency index to model energy consumption - a critical factor for determining a robot’s potential operational longevity.

#### Setup factor importance

To assess how important participants believed each of the seven different setup factors were in supporting a sense of presence and a performance-focused task, they rated each factor in these two aspects on a 10-point scale (where 1 is not at all important, 5 is neutral, 10 is very important).

### Virtual environment

**Fig. 2:**
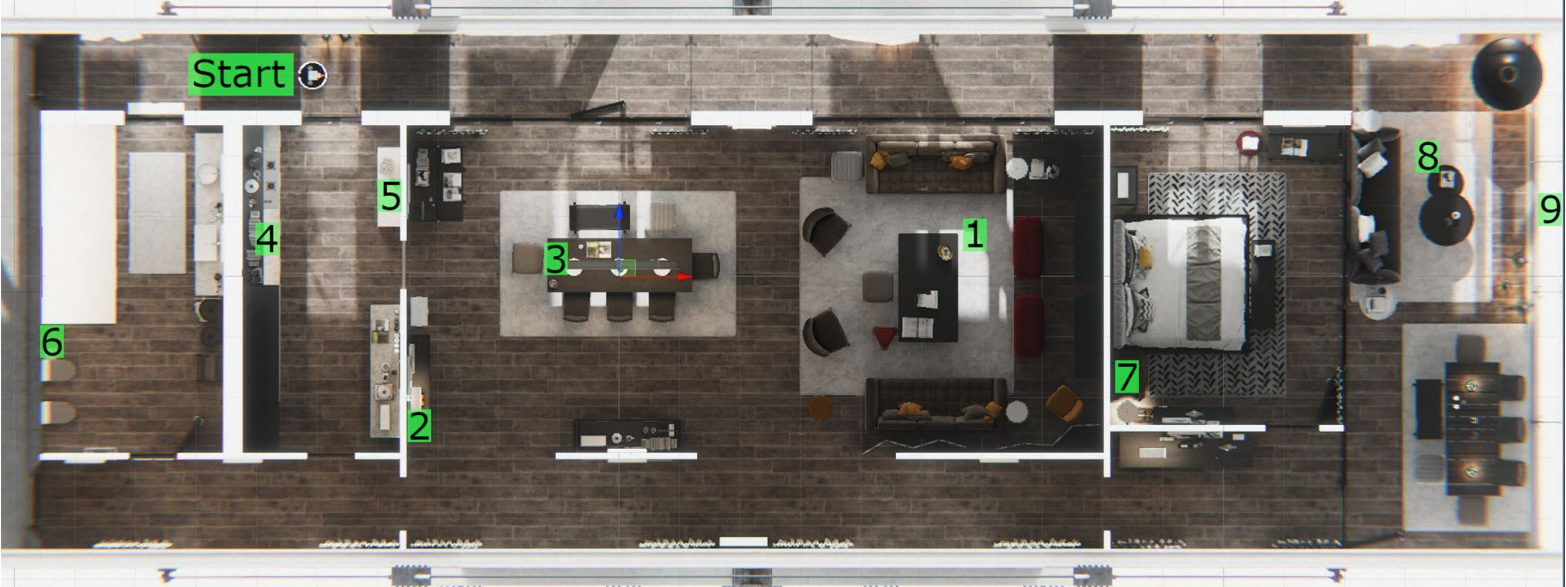
Virtual Environment and Study Tasks. Overhead view of the virtual environment of the home used in the study. The robot’s starting position, in the top left, is labeled. Each of the 9 task locations are also labeled in the order they were to be completed

Participants controlled a simulated telepresence robot in a VE of a one-story home with multiple areas (balcony, living room, kitchen, bathroom, hallways, and bedroom) (Figure 2). This environment was chosen because of its high quality, realism, and obstacle layout (see Supplementary material 2 for a video showing the VE). To motivate participants and evaluate their use of each setup on their maneuvering performance (i.e., obstacle avoidance) under time pressure, we presented them a fictitious story where they worked as an inspector for a short-term home rental service (i.e., AirBNB) and had to use a telepresence robot to inspect a home, in a remote location, between rentals. This task is based on a real-world use of telepresence robots to remotely check in on your home (Yang and Neustaedter 2018; Hung et al. 2022).

### Study task

Participants were tasked with maneuvering to specific locations and answering questions about the objects located there (i.e., number of keys inside the dining table’s bowl or what was left behind the bedroom shelf). Participants were instructed to complete search tasks as quickly as possible while minimizing collisions with static obstacles (walls and furniture). See Supplementary material 2 for a video showing the task participants performed.

Because the goal was to investigate how learnability differed between the two setups and to focus on maneuvering rather than navigation, the path participants had to traverse was fixed, as were the task locations and order. Participants were shown the route beforehand to eliminate any path-planning/finding. They were instructed to follow this route on each trial, as this was the order the search tasks were presented. To increase engagement, search task objects were randomized, with three possible answers per task. To verify participants went around the home, their answers were checked. Note that to answer questions, a keyboard and mouse were used for the Standard setup and the tablet’s touchscreen for the Novel setup. The time spent answering questions was not included in the statistical analyses (i.e., task completion and collision time analyses).

Determined through pilot testing as a way to motivate participants to maneuver across the home while providing a task difficult and engaging enough to evaluate the participants’ use of the setups, each trial consisted of an inspection with 9 objects placed around the home (Figure 2). Five objects required participants to look at the down-facing camera (e.g., look into a sink or bowl to see what was inside), while the others required them to look at the forward-facing camera (e.g., read the name of a book on a high shelf or count how many snowmen were outside the home); this allowed for some variability across tasks and required participants to use both camera views. To make use of the setups’ audio output and help immerse participants in the VE (by masking out sounds of the real-world environment by playing sounds from the VE), ambient music from the home’s living room was played.

### Equipment

The VE was created using the Unity Game Engine version 2020.3 and rendered on a laptop (NVMe Micron 2230 SSD, Intel i7-10875 H @ 2.30GHz, 32GB DDR 4 RAM, NVIDIA RTX 2080 SUPER Max-Q 8GB GDDR 6, 15.6-inch Display, and Resolution of 1920 x 1080 pixels). This environment was displayed on the laptop’s monitor, for both setups, and also on a tablet, an iPad Pro (6th Generation, Apple M2, 8GB RAM, 12.9-inch Display, and Resolution of 1440 x 1080 pixels), as a secondary screen for the Novel setup. Audio was played on the laptop’s speakers, in the Standard setup, and via a wired Sony noise-canceling headphones (WH-1000XM3), in the Novel setup. The IMU, used in the Novel setup to detect the rotation of the chair, was the PhidgetSpatial Precision 3/3/3 High-Resolution model 1044_0. The joystick, used in the Novel setup for translation input, was the Logitech G Freedom 2.4 GHz Cordless Joystick.

### Telepresence robot simulation

The simulated telepresence robot system is a modified version of the one used in (Suomalainen et al. 2022): the virtual robot is modeled after the GoBe telepresence robot (the follow-up to the popular Beam model), a balanced differential drive robot with the 2 driving wheels in the middle and 4 caster wheels in corners to keep it steady, essentially a nonholonomic system that can rotate in place, translate forward and backward with and without rotating at the same time (curved or straight paths), but cannot move sideways (strafe). Although telepresence robots with omnidirectional navigation do exist, allowing translation in all directions as well as rotation in place and on a curve, they are not widely used commercially (Torrejón et al. 2024) and are usually found on robots designed for research purposes (Ferland et al. 2013; Torrejón et al. 2024) or competitions (Hauser et al. 2025). Since omnidirectional navigation would require hardware modifications to typical telepresence robots, we opted for a DDR-type of movement for our robot.

To control the robot’s rotation for the Novel setup, a proportional-integral-derivative (PID) controller (with Kp, Ki, and Kd values of one) was implemented and it had as input the yaw angle difference between the chair (IMU) and the robot. We chose to use a simulated telepresence robot and home environment because simulation has been shown to reduce experimental costs compared to using real robots, allow users to test robots without having one physically available (Rehman et al. 2013; Román-Ibáñez et al. 2018), improve and accelerate robot prototyping (Bogaerts et al. 2020; Rohmer et al. 2013; Wachs et al. 2005), allow users to practice and become familiar with controlling robots without risk of injury (Chen et al. 2013), support human-robot interaction studies (Liu et al. 2017; Wijnen et al. 2020; Dole and Ju 2019), study robot use when real-world scenarios are dangerous or impractical (Stallings 2007; Murphy 2017; Agüero et al. 2015; Kanehiro et al. 2019), and the knowledge gained in a simulated environment can be transferred to a real-world environment (Villani et al. 2018) (see (Collins 2022; Collins et al. 2021; Kargar et al. 2024) for more information on the use of robot simulators). This allowed us to quickly develop and test different setup and locomotion iterations and without having to worry about collisions damaging a real robot or its environment.

The system’s values for our simulated robot were initially based used on the approximate values used in (Suomalainen et al. 2022), which had a maximum speed of approximately 60 deg/s and the camera was set at a height of 1.5 m above its base. Through pilot testing these values, we came to values that best fit our VE while ensuring realistic measures and task difficulty. Our simulated robot had a cylindrical base with a height of 2.8 cm and a diameter of 45 cm, roughly matching the base dimensions of the commercial telepresence robots GoBe (45.5 x 52.5 cm) and Beam Pro (50.8 x 66 cm). Its maximum translation speed was set to 1 m/s, similar to typical commercial robots such as the GoBe (0.9 m/s). Rotation speeds were limited to 60 deg/s, aligned with (Suomalainen et al. 2022) and only slightly faster than the 36 deg/s of the Double 3 telepresence robot. The forward and down-facing cameras were both at a height of 1.52 m above ground, roughly matching the height of (Suomalainen et al. 2022) (1.5 m) and the GoBe robot (1.3 m). The forward and down-facing cameras had vertical and horizontal fields of view of 70 and 102.45 degrees and 70 and 86.07 degrees, respectively. The down-facing camera had a pitch rotation of 70 degrees downward, in relation to the forward-facing camera. As shown in Figure 1, for the Standard setup, the forward and down-facing camera views were displayed in frames of $960 \times 540$ and $680 \times 510$ pixels in size, respectively. These camera view sizes were established as a result of pilot tests and from calculations considering the size and resolution of the laptop’s monitor and tablet and the distance between them. For the Novel setup, the forward and down-facing camera views were displayed in full-screen on the laptop’s monitor at a resolution of $1920 \times 1080$ pixels and tablet at a resolution of $1440 \times 1080$ pixels.

### Participants

We recruited 42 participants (22 male, 19 female, 1 preferred not to say) for this study, whose ages ranged from 19 to 42 ($M = 24.62, SD = 6.01$). 23 participants ($60\%$) had corrected eyesight (glasses or contact lenses). Of the 42 participants, five of them had their data either partially excluded from data analysis due to problems that occurred during trials (i.e., sounds occurred in or around the lab that made participants nervous) or fully excluded (due to power failure or the participant did not follow instructions). This study had the approval of the local Research Ethics Board ($\#20180649$) and participants, those who were students, were compensated with course credit for their time.

### Procedure

**Fig. 3:**
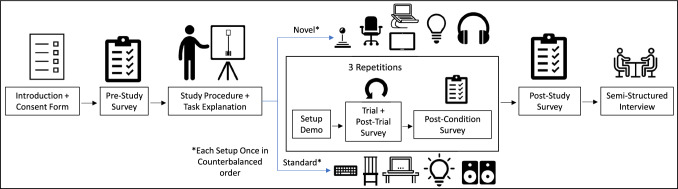
Study Procedure. The figure shows an overview of the study’s procedure. The main steps were the introduction and initial forms, study and setup explanations, study trials and surveys, and post-study survey and interview

The procedure used in our study (illustrated in Figure 3) began with an introduction of the researcher to the participant and having the participant complete a consent form and a pre-study survey. The researcher then explained the study procedure and the task the participant had to perform. The first setup the participant would use, determined via counterbalanced study order, was then explained and demonstrated. The participant then performed the study task, which took around three minutes. After each trial, participants completed a post-trial survey consisting of a Simulator Sickness Questionnaire (SSQ) (Kennedy et al. 1993), to measure visually-induced motion sickness (VIMS) (Bos et al. 2008); a NASA-Task Load indeX (TLX) questionnaire (Hart et al. 1988), to measure perceived workload; a question about how strongly they felt vection (the illusion of self-motion in an environment in the absence of physical motion (George and Fitzpatrick 2011; Palmisano et al. 2015)); and a question about how aware they were of their real environment (presence). After completing all 3 trials with one setup, and the post-trial survey each time, participants answered a post-condition questionnaire consisting of introspective user experience, usability, and presence/spatial presence questions (considering all or a subset of items in the Igroup Presence Questionnaire (IPQ) (Schubert et al. 2001)) about the setup they just used. Participants would then repeat this process for the second setup. After completing the post-condition survey with both setups, participants answered a post-study survey with questions directly comparing each setup (presence, ease of control, which setup better suited different use cases, and rated the importance of setup factors in terms of presence and performance). We then conducted a semi-structured interview, which took around 25 min, with open-ended questions with participants to gather their thoughts on using the setups to complete tasks and their individual factors. The total study time was around an hour and fifteen minutes.

## Results

Inferential statistical analysis on the objective and subjective measures were conducted using $2\times 3$ repeated measures ANOVAs with setup (Novel vs. Standard) and trial (first, second, third) as independent variables. Planned contrasts (ANOVA test slices) were used to better understand the nature of significant interactions. The introspective user experience, usability, and use case setup suitability measures violated the normality assumption and were therefore analyzed using the Wilcoxon signed-rank test (non-parametric repeated measures t-test). The remaining ordinal data, comparing setups on ease of control and sense of presence, were analyzed using Pearson’s Chi-Squared test. Results were analyzed using the JMP statistical program (version 16) with (two-tailed) alpha levels set at 0.05 ($p \le .05$, statistically significant) and 0.10 ($p \le .1$, marginally statistically significant) and a $95\%$ confidence interval.

### Presence and vection measures

**Table 1:**
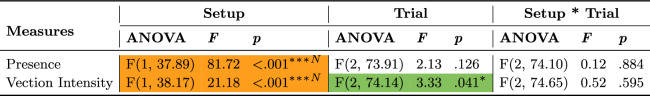
ANOVA results for subjective Presence and Vection Intensity measures across trials.

Statistically significant (* $p \le .05$ and *** $p \le .001$) effects are highlighted in a darker shade. Orange and “N” indicate the Novel setup’s advantage over the Standard while green indicates a general effect not necessarily favoring any setup

**Table 2:**
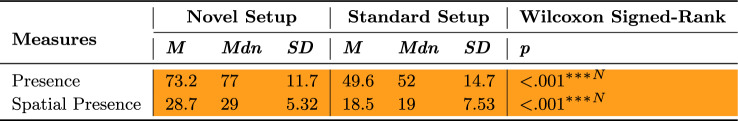
Wilcoxon Signed-Rank Test for subjective Presence and Spatial Presence measures comparing setups.

Statistically significant ($p \le .001$) effects are highlighted in a darker shade. Orange and “N” indicate the Novel setup’s advantage over the Standard setup

**Table 3:**
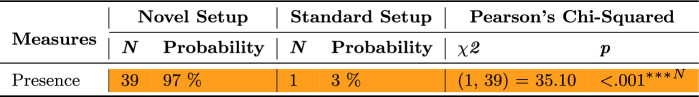
Pearson’s Chi-Squared Test for the subjective Presence measure comparing setups.

Statistically significant ($p \le .001$) effect is highlighted in a darker shade. Orange and ''N'' indicate the Novel setup’s advantage over the Standard setup

**Fig. 4:**
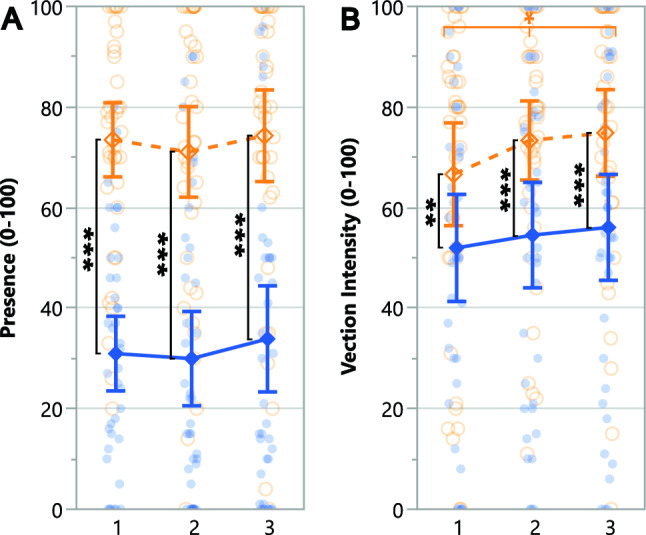
Pairwise planned contrast results for the Presence and Vection intensity measures across trials for the Novel setup(dashed orange lines) and Standard setup (solid blue lines). Error bars represent confidence intervals ($CI = 95\%$) and dots show data of individual participants for the Novel setup (empty orange circles) and Standard setup (filled blue circles). Along the top of the graph, depicted via horizontal lines, asterisk annotations represent statistical significance across all trials (a change over time/learning effect) for a setup. Inside the graph, depicted via vertical lines, asterisk annotations indicate statistical significance between setups for a given trial (* $p \le .05$, ** $p \le .01$, *** $p \le .001$)

Overall presence, spatial presence, and vection intensity measures are summarized in tables 1, 2, and 3. Presence and vection intensity measures are plotted in Figure 4. Participants felt a significantly stronger sense of being in the displayed environment (presence) and moving through it (vection intensity) with the Novel setup compared to the Standard (Table 1). Planned contrasts revealed that participants felt a greater sense of presence (Figure 4A) and vection (Figure 4B) with the Novel setup compared to the Standard across trials. Participants felt a greater sense of presence and spatial presence when using the Novel setup (Table 2), and they chose it as the setup that provided a greater sense of presence (Table 3).

### User experience

Six measures of user experience are summarized in Table 4. Participants felt the Standard setup was less tiring, with muscles more relaxed, and more comfortable, while they felt a greater sense of engagement, enjoyment, and excitement when using the Novel setup.

**Table 4:**
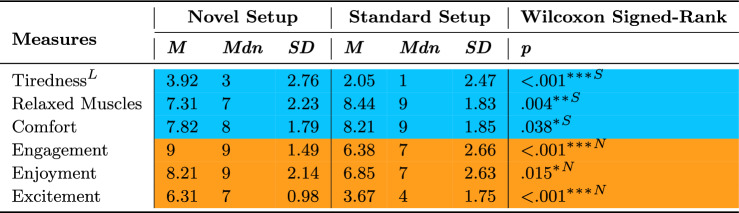
Wilcoxon Signed-Rank Test for the subjective User Experience measures comparing setups.

Statistically significant (* $p \le .05$, ** $p \le .01$, *** $p \le .001$) effects are highlighted in orange with an ”N” symbol, favoring the Novel setup, and in blue with an ”S” symbol, favoring the Standard setup The "L" symbol represents measures where lower values are better

#### Motion sickness (SSQ)

**Fig. 5:**
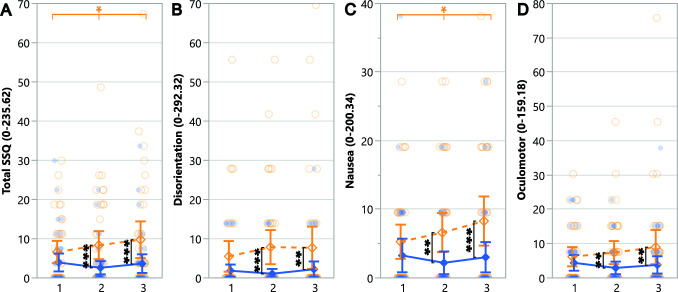
Pairwise planned contrast results for the SSQ measure and its sub-components across trials for the Novel setup(dashed orange lines) and Standard setup (solid blue lines). Error bars represent confidence intervals ($CI = 95\%$) and dots show data of individual participants for the Novel setup (empty orange circles) and Standard setup (filled blue circles). Along the top of the graph, depicted via horizontal lines, asterisk annotations represent statistical significance across all trials (a change over time/learning effect) for a setup. Inside the graph, depicted via vertical lines, asterisk annotations indicate statistical significance between setups for a given trial (* $p \le .05$, ** $p \le .01$, *** $p \le .001$)

Motion sickness measures (Total SSQ and its three components - disorientation, nausea, and oculomotor issues) are summarized in Table 5 and plotted in Figure 5. Participants experienced significantly more motion sickness when using the Novel setup compared to the Standard setup. Planned contrasts revealed that although participants experienced similar levels of motion sickness (SSQ total and subscores) with both setups in the first trial, they experienced more motion sickness with the Novel setup in later trials and compared to the Standard setup, indicating worsening effects from using the Novel setup over time. Although this occurred, the highest mean value for the Novel setup (trial three) was 9.74, representing only 4.13% of the SSQ scale (ranging from 0 to 235.62). Furthermore, no participant complained of motion sickness between trials or wanted to stop the study.

**Table 5:**
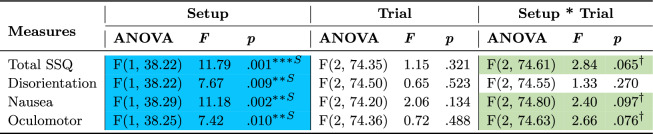
ANOVA results for the subjective SSQ measure and its sub-components comparing setups across trials.

Statistically significant (** $p \le .01$ and *** $p \le .001$) effects are highlighted in a darker shade and marginally significant ($\dag$ $p \le .1$) effects are highlighted in a lighter shade. Blue and ”S” indicate the Standard setup’s advantage over the Novel while green indicates a general effect not necessarily favoring any setup

### Usability

Fourteen measures of usability are summarized in tables 6 and 7. Participants felt the Standard setup was safer, they would use it more regularly and for longer periods of time (Table 6), and that it was easier to control (Table 7) than the Novel setup. In contrast, participants perceived the Novel setup to be significantly less complicated and confusing to use and that it allowed for more precise control.

**Table 6:**
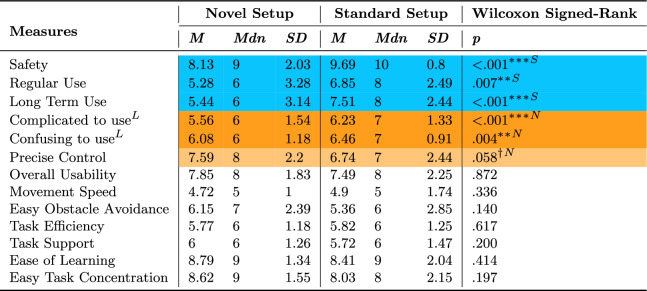
Wilcoxon Signed-Rank Test for thirteen subjective Usability measures comparing setups.

Statistically significant (** $p \le .01$, *** $p \le .001$) and marginally significant ($\dag$ $p \le .1$) effects are highlighted in orange and lightorange with an ”N” symbol, favoring the Novel setup, respectively, and in blue with an ”S” symbol, favoring the Standardsetup The "L" symbol represents measures where lower values are better

**Table 7:**
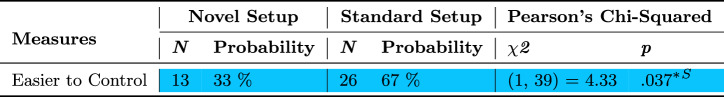
Pearson’s Chi-Squared Test for the subjective Ease of Use Usability measure comparing setups.

Statistically significant (* $p \le .05$) effect is highlighted in a darker shade. Blue with an ”S” symbol, favoring the Standard setup

#### Task load

**Fig. 6:**
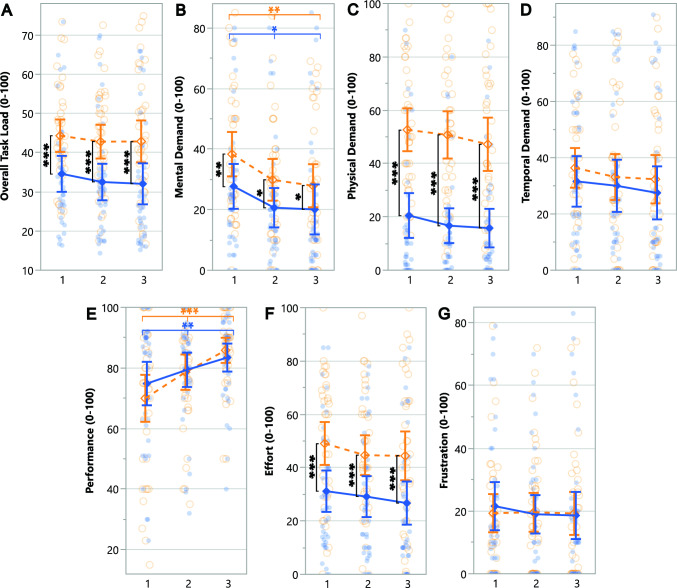
Pairwise planned contrast results for the Task Load measures across trials for the Novel setup (dashed orangelines) and Standard setup (solid blue lines). Error bars represent confidence intervals ($CI = 95\%$) and dots show data of individual participants for the Novel setup (empty orange circles) and Standard setup (filled blue circles). Along the top of the graph, depicted via horizontal lines, asterisk annotations represent statistical significance across all trials (a change over time/learning effect) for a setup. Inside the graph, depicted via vertical lines, asterisk annotations indicate statistical significance between setups for a given trial (* $p \le .05$, ** $p \le .01$, *** $p \le .001$)

Task load, divided into seven sub-measures, are summarized in Table 8 and plotted in Figure 6. Participants perceived significantly greater overall task load (final weighted score), physical demand, effort, mental demand, and marginally greater temporal demand when using the Novel setup than when using the Standard setup. The setups had similar results for rated performance and frustration.

Planned contrasts revealed that participants felt greater levels of overall task load (Figure 6A), physical demand (Figure 6C), and effort (Figure 6F) when using the Novel setup compared to the Standard setup overall, which did not change across trials. Although the mental demands of using each setup and the difference in demands between the setups decreased throughout trials (Figure 6B), participants perceived the Standard setup as less mentally demanding. Furthermore, although participants perceived that their performance improved across trials for both setups (Figure 6E), more so for the Novel setup, there was no perceived performance difference between the setups. Moreover, participants perceived similar levels of temporal demand (Figure 6D) and frustration (Figure 6G) between the setups throughout trials.

**Table 8:**
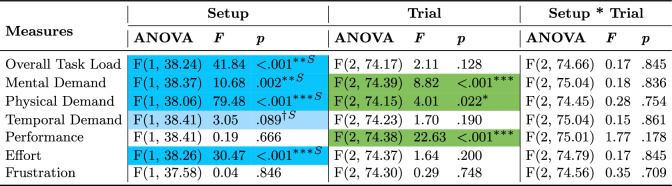
ANOVA results for subjective Task Load measures comparing setups across trials.

Statistically significant (* $p \le .05$, ** $p \le .01$, *** $p \le .001$) effects are highlighted in a darker shade and marginally significant ($\dag$ $p \le .1$) effects are highlighted in a lighter shade. Blue and ”S” indicate the Standard setup’s advantage over the Novel while green indicates a general effect not necessarily favoring any setup

#### Use case setup suitability

Although our study’s task was performance-focused, we asked participants to imagine which setup they would prefer to use in both performance and social-focused situations, as summarized in Table 9. Participants rated the Novel setup as more suitable for travel or sightseeing, personal social situations, and both small and large professional gatherings compared to the Standard setup.

**Table 9:**
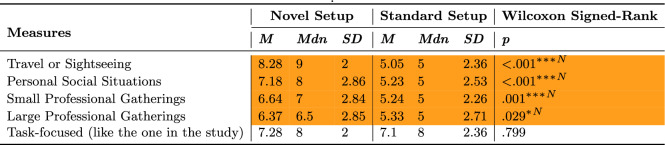
Wilcoxon Signed-Rank Test for the subjective setup suitability on different use cases between setups.

Statistically significant (* $p \le .05$ and *** $p \le .001$) effects are highlighted in orange with an ”N” symbol, favoring the Novel setup

### Performance

**Fig. 7:**
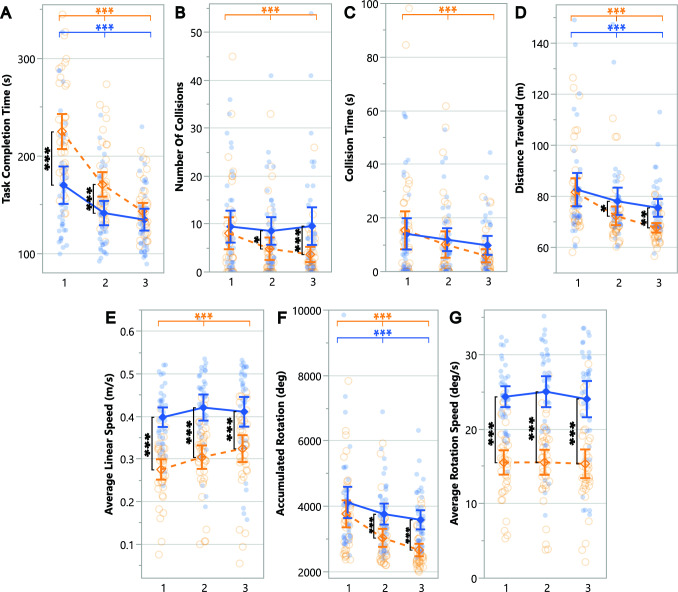
Pairwise planned contrast results for the Behavioral measures across trials for the Novel setup (dashed orangelines) and Standard setup (solid blue lines). Error bars represent confidence intervals ($CI = 95\%$) and dots show data of individual participants for the Novel setup (empty orange circles) and Standard setup (filled blue circles). Along the top of the graph, depicted via horizontal lines, asterisk annotations represent statistical significance across all trials (a change over time/learning effect) for a setup. Inside the graph, depicted via vertical lines, asterisk annotations indicate statistical significance between setups for a given trial (* $p \le .05$, ** $p \le .01$, *** $p \le .001$)

Seven performance measures are summarized in Table 10 and plotted in Figure 7. Participants significantly improved their performance when using the Novel setup compared to the Standard setup, as indicated by a reduction in the number of collisions, distance traveled, and accumulated rotations. Conversely, using the Standard setup resulted in increased average linear and rotational speeds. Planned contrasts were used to further investigate results and their findings are presented below.

**Table 10:**
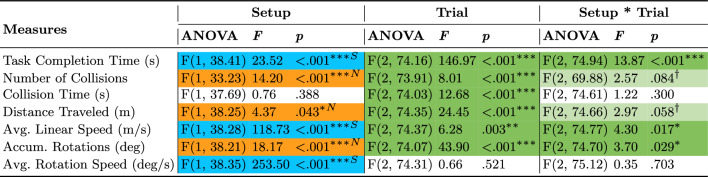
ANOVA results for the objective Behavioral measures comparing setups across trials.

Statistically significant (* $p \le .05$, ** $p \le .01$, *** $p \le .001$) and marginally significant ($\dag$ $p \le .1$) effects are highlighted in green and light green, respectively

#### Task completion time

Participants completed tasks faster with the Standard setup in the first two trials and they improved with both setups across trials, decreasing their time (Figure 7A). By the third trial, participants performed similarly with both setups, indicating greater learning effects for the Novel setup.

#### Distance traveled and accumulated rotation

Participants traveled (Figure 7D) and rotated (Figure 7F) less with both setups across trials. Although participants performed similarly on these measures with both setups on the first trial, they performed better (traveled and rotated less) with the Novel setup in subsequent trials, indicating a stronger learning effect for the Novel setup.

#### Collision

Although participants collided with a similar number of objects on the first trial with both setups (Figure 7B), they collided less with the Novel setup across trials and in comparison to the Standard setup. Consequently, participants spent less time colliding with objects across trials with the Novel setup (Figure 7C), indicating a learning effect for the Novel setup. Although collision times decreased for the Novel setup, they were still similar between setups across trials.

#### Average linear and rotation speeds

When using the Novel setup, participants experienced an increase in their average linear speed (Figure 7E) while their average rotation speed (Figure 7G) did not change across trials. When using the Standard setup, participants maintained similar linear and rotation speeds across trials. Participants moved and rotated faster with the Standard compared to the Novel setup across all trials.

### Efficiency indexes

**Fig. 8:**
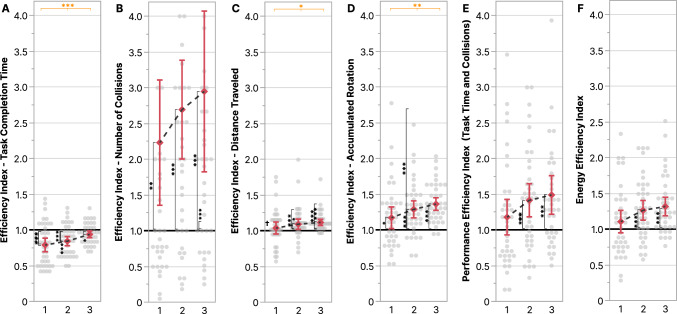
Pairwise planned contrast results for the Efficiency Indexes across trials. Error bars represent confidence intervals ($CI = 95\%$) and dots show normalized data of individual participants. Along the top of the graph, depicted via horizontal lines, asterisk annotations represent statistical significance across all trials (a change over time/learning effect). Inside the graph, depicted via vertical lines, asterisk annotations indicate statistical significance (* $p \le .05$, ** $p \le .01$, *** $p \le .001$) between the normalized value mean and 1 (no difference between setups), depicted via a continuous line

To analyze the change in efficiency across trials, a one-way ANOVA was used and its results were summarized in Table 11 and plotted in Figure 8. Results showed that task completion time, distance traveled, and accumulated rotations efficiencies significantly changed across trials. To analyze which of the setups was more efficient in each trial, t-tests were used to compare the efficiency indexes’ mean values to a value of 1, results were summarized in Table 12, plotted in Figure 8, and presented in more detail below.

#### Task Completion Time

Though the Standard setup was more efficient in task completion time throughout all trials (Figure 8A), there was a significant decrease in this efficiency from 21% to 6% as participants improved more throughout trials with the Novel setup.

#### Number of collisions, distance traveled, and accumulated rotation

The Novel setup was more efficient in terms of number of collisions (Figure 8B), distance traveled (Figure 8C), and accumulated rotations (Figure 8D). These efficiencies increased further; by the third trial, participants collided less (195%), traveled less (12%), and rotated less (36%) compared to the Standard setup.

#### Performance and energy

Though participants had similar performance (Figure 8E) and energy efficiencies (Figure 8F) in the first trial, they improved with the Novel setup over time such that they were 49% more performant and 32% more energy efficient by the end of the third trial.

**Table 11:**
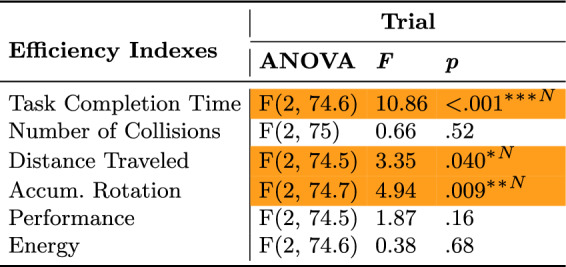
ANOVA results for the objective Efficiency Indexes across trials.

Statistically significant (* $p \le .05$, ** $p \le .01$, and *** $p \le .001$) effects are highlighted in a dark shade. Orange with an ”N” symbol indicates an advantage for theNovel setup and blue with an ”S” symbol indicates an advantage for the Standard setup.

**Table 12:**
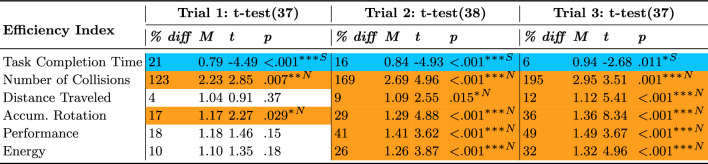
T-test results for the objective Efficiency Indexes across for each trial.

Statistically significant (* $p \le .05$, ** $p \le .01$, and *** $p \le .001$) effects are highlighted in a dark shade. Orange with an ”N” symbol indicates an advantage forthe Novel setup and blue with an ”S” symbol indicates an advantage for the Standard setup.

### Setup factor importance rating

As detailed in Table 13, participants rated lighting, audio output, and rotation method as the three most important factors for supporting a sense of presence, while rotation method, translation method, and display were rated as the three most important factors for improving performance.

**Table 13:**
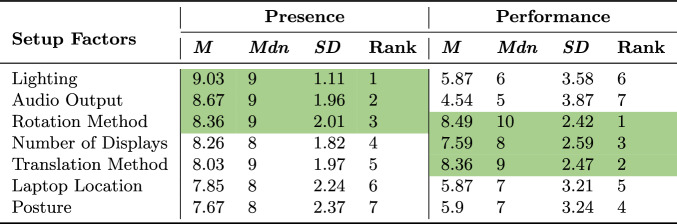
Subjective ratings on how important each setup factor was to participants in supporting presence and task performance. The rank column depicts the order mean rating of each factor’s perceived importance. The top 3 factors for presence and performance are highlighted in green

## Interview observations

This section aims to provide a more nuanced understanding of the user experience of the Novel and Standard setups by exploring the "why" behind the quantitative results and highlighting key topics that emerged from observations and comments participants made during their interview. The interviews were semi-structured with open-ended questions that focused on gaining insight into participants’ overall thoughts about using each setup to perform tasks. Questions also explored the experience of controlling the robot, particularly while navigating the home and avoiding obstacles, and sought to identify which setup participants preferred for the task and the importance of their factors. Additionally, questions sought whether participants experienced motion sickness and, if so, what they thought were possible causes. Questions also investigated the types of scenarios participants believed they would prefer to use each setup in and the reasons for their choices. Finally, questions encouraged participants to suggest improvements to the setups and share their vision of an ideal setup. Interviews were audio recorded and transcribed. One of the researchers went through the data and used affinity diagrams to make sense of, group, and organize data by topic similarity (Hartson and Pyla 2012; Kawakita 1991; Holtzblatt et al. 2004). “Px” represents the participant number attributed to a quote, i.e., P1 means Participant 1.

### Presence and immersion

Participants expressed a greater sense of presence with the Novel setup, exemplified by [P7] describing their feeling of presence with the Novel setup as *"I was 100% immersed in that world, [...] you transport yourself to that world"*, and that this feeling increased over time [P5,7], described by [P5] as *"the second time you get more and more immersed in that world [...] you get carried away so much that the third time it seemed like a door opened and I entered this world."*. Participants suppose this is due to **immersion from minimized distractions** (lights off [P8,11,12,18] and headphones use [P8,12,29,36]); screen size, placement, and FoV [P5,12,13,36]; better posture [P7,13,36]; and sense of **embodiment** with joystick [P7,21] and chair rotation [P7,13,21,27,36]. [P36] believed that physically rotating made them feel so much like the robot that they would unconsciously tuck in their arms to not hit obstacles while maneuvering, yet with the Standard setup they didn’t feel like they *"were there"*. With the Standard setup, participants were aware of their real-world surroundings due to lights being on [P1,11] and use of speakers, since they could still see and hear their surroundings [P12,28,38]. [P36] felt that the Novel setup’s high demands caused them to better focus.

Participants mentioned an importance of having the appropriate ***"mindset"*** in order to become immersed. [P11,13,21] felt less immersed with the Standard setup because they were ***“conditioned”*** to associate sitting at a desk and having the lights on to performing a non-immersive task on a computer. Whereas with the Novel setup, due to differences in **appearance** and embodiment and immersion aspects between the setups, [P21] felt they were in *"a cockpit”* and [P13] like it was VR, this put them in a different mindset that allowed them to become immersed.

Participants [P11,21] discussed the **importance of feeling present**, they felt more **integrated** with their task, **careful**, and **cared more about** the consequences of their actions, especially in tasks that do not allow for errors. For instance, [P7] described feeling present with the Novel setup allowed them to *“dodge more easily [...] I felt 100% in control, like it [the robot] was my body”*, performing better than with the Standard setup’s keyboard.

### User experience and usability

#### Motion Sickness

Participants [P3,23,27,29,37,42] noted symptoms of **motion sickness**, especially dizziness, and believed it was because of them spinning in the chair. Participants [P3,8] also mentioned eyestrain, a symptom of motion sickness, due to focusing on bright screens in the dark. Regarding the Standard setup, participants [P2,23] also mentioned feeling dizzy, which they suppose is because of the camera views, which were too close to each other while having different viewing angles, something they did not feel with the Novel setup.

#### Setup physical and cognitive demands

Though some participants thought the setups had similar levels of low physical demand [P1,3,6,7,26,42], most found the Novel setup more tiring, requiring more effort and physical demand as they had to use their legs to rotate [P8,12-14,27,28,32,36,40], stating that they sweated [P11] and it ***"felt like exercise"*** [P10,21], which [P10] enjoyed as they had to exercise anyway. [P40] believed the chair’s **heaviness** was due to the equipment on it. Participants also felt tired [P22] and uncomfortable [P36] due to head movements required to look at the Novel setup’s screens, as opposed to eye movements with the Standard setup. In contrast, [P36] felt less tired because of the higher physical and cognitive demands, causing them to feel more awake and focused on what they were doing.

Regarding the Standard setup, participants [P8,27,40] felt it to be less tiring and physically demanding due to using their fingers to press keys on a keyboard. [P36] felt low physical and mental demands with the Standard setup, as it felt like playing a video game.

#### Long term use

Participants believed they would feel tired from using the Novel setup for long periods due to using their legs to rotate [P17,18,21,23], and for the Standard setup from using their fingers [P17] or from sitting in the same posture [P28]. Participants felt they could use the Novel setup for longer periods of time because of how immersed and entertained they felt [P7], where the duration is task-dependent (how much movement and looking around it requires) [P27]. Other participants would prefer the Standard setup, as they hypothesized that they might get too motion sick with the Novel setup over time [P3,16,23,24,27].

#### Comfort

Some participants [P1,3,23,26,30] felt similar comfort levels between setups, while others preferred the Standard setup because it was less physically demanding [P22] or because it allowed them to quickly and easily get out of the chair [P35]. The remaining participants that discussed comfort preferred the Novel setup because they could sit in a leaned-back and **relaxed posture** [P5,7,9,11,27,13-15,20,24,25,28,36]; easily visualize the displayed environment, due to screen position and size, without moving close to screens [P8,10,13,15,23,25]; freely move/rotate around [P28,36]; and feel more in control, due to the precise input [P10,18,28].

#### Safety

Though participants mentioned feeling safe with both setups [P13,18,28,32], others expressed concerns with the Novel setup because they were anxious when they heard a sound from the real world and could not see what it was [P3,10], or worried about rotating in the dark and hitting something/someone around them [P22,29], especially at home with kids [P18], paying more attention to the real world with this setup. Participants [p16,27] were concerned with the Novel setup’s prototypical appearance, that something could fall off, making them cautious when going very fast with the robot [P27], as opposed to the Standard which had less factors to worry about and they believed people would find safer. [P17] had initial concerns about the Novel setup’s safety, but felt safe after a few minutes of using it.

#### Intuitive, easy to learn, and easy to control

Participants [P12,16,19] thought the Novel setup, especially the input methods, would be difficult and time-consuming to learn, but they found it quick and easy to learn, taking only minutes [P12,35]. They improved over time, such that they could move faster than with the Standard setup [P19]. Participants felt that physical rotation was **natural** and realistic, as body movements translated to them moving in the environment [P7,13,26]. Participants [P8,19,23] felt that the Novel setup’s screen positions were intuitive, mimicking how you look around in real life, looking forward and downward to view those directions, while the Standard screens felt too condensed and confusing. [P13] felt the setup was like VR but without a VR headset, they could intuitively look around to view the environment.

Participants felt that using the joystick to translate [P12,23,31,33] and their body to rotate [P12,19] gave them more **precision**, allowing them to go faster or slower at their own pace, which was especially helpful for maneuvering around corners and making small adjustments. They felt they lacked control with the keyboard, even when using a *"feathering"* technique (quick consecutive taps) [P12], because it was always fast, making it imprecise, difficult to use, and causing overshoots which required corrections [P19]. Participants [P3,11,26,27] found the Standard setup easy to learn due to **familiarity** with the keyboard, especially those with gaming experience [P13,21,35,36], which is a reason why some participants preferred to use this setup. Participants [P31,40] expressed a lack of control with the joystick and chair rotation because it was difficult to reach maximum speed as opposed to simply pressing a key on a keyboard, causing them to feel frustrated and hit more obstacles.

#### Engagement, enjoyment, and excitement

Participants [P21,27,33] described the Standard setup as monotonous and boring, like a computer task/chore they had to perform, while the Novel made even repetitive tasks **pleasurable** and **exciting** as it was something new (i.e., using physical movement to rotate). [P27] felt as if they rotated with the robot when using the Novel setup, making it more enjoyable than the Standard setup, even if it induced motion sickness.

### Task performance

Participants [P2,7] expressed a lack of familiarity with the robot’s body size as opposed to their own body, requiring them to look at the bottom view more often than the forward view to avoid obstacles, something they don’t do when walking in the real world. Participants also mentioned that the Novel setup’s larger screen size, wider FoV, and position, referring to the bottom screen as if it were **peripheral vision** [P7], positively impacted their performance, making it easier to see things [P7,13] than with the Standard setup. [P36] felt the Standard setup allowed them to make quick eye movements to see both camera views, making maneuvering easier, but had difficulty seeing objects on the screens, requiring them to lean in and squint. While the Novel setup required uncomfortable head movements to view screens, they did not have to lean in or squint which improved their comfort and search for objects. [P7,21] noted that repeated use improved performance with the Novel setup and more **repetitions** would further improve performance *“more times will lead to perfection.”*

### Use cases

#### Social Scenarios

[P21] believed that using the Novel setup in social environments will **elicit engagement and empathy** from others towards them as a robot, because others will notice that their movements were made from their body rather than from a keyboard. Some participants would prefer the Novel setup in social situations because they believe it would make them *"feel more present when talking to people"* [P5,12,27] and focused [P12,33], whereas the Standard setup would simply feel *"like a zoom video"* [P5,26] and not immersive.

Other participants would prefer to use the Standard setup in social situations because they felt better control and would bump into people less [P3], and would like to quickly turn around when someone comes up behind them to talk (180-degree rotation), where a keyboard would rotate them faster than physically rotating around [P40]. [P36] would prefer the Standard setup because they believe using the Novel setup would make them feel like they are playing a game or having too much fun, which would not be appropriate for a work environment.

### Suggestions for improvements

Participants provided diverse insights for further improving the Novel setup:

***Locomotion Method:*** Some participants suggested adjusting rotation gains to reduce the amount of physical rotation required [P1,6,34,37]. Others suggested controlling rotations via steering wheel or pedals [P1,3,22], joystick [P2,40], mouse [P2,40], or gamepad [P40]. A participant suggested using a motorized chair and controlling its rotation via joystick [P12]. Participants also suggested incorporating strafing (sideways movement) and using a gamepad to improve maneuverability ([P13,40]), and using a modifier (i.e., a button or key press/hold) to increase or decrease speeds [P23].

***Presence, Immersion, Spatial Awareness, and Display:*** To further enhance presence, immersion, and spatial awareness, participants suggested using a wide curved screen [P8,23], VR HMD [P13,23,36,40], 360-degree screens coupled with an omnidirectional treadmill [P22,33], or adding directional audio playback [P6,40].

**Collision Avoidance:** Participants suggested various collision avoidance features, such as a rear/back camera [P29,31], proximity sensors with on-screen visuals [P21,31], haptic feedback [P5,21,40], or audio cues [P5,21]. Other suggestions included shared control to decrease speed based on proximity [P23,40], emergency stop [P36], and padding around the robot to minimize damage in case of collision [P36].

***Mixing Elements and Providing Choice:*** Finally, participants suggested mixing elements from the Standard and Novel setups, such as using the Standard setup with the Novel’s displays [P9] or laptop stand [P7], using the Novel setup with a keyboard for maneuvering [P9,23,40], or providing the option to choose (and change during use) input methods, such as physical vs. keyboard/joystick rotation [P8].

While many of the participants’ suggestions are feasible and could improve the system, some, such as incorporating 360-degree screens or an omnidirectional treadmill, may be impractical or too expensive for most applications. Future research could explore how these diverse suggestions could be synthesized into a new and improved telepresence robot setup.

## Discussion

We found that the Novel setup, which is the result of using simple methods and tools to modify a typical telepresence robot operator interface (the Standard Setup) without making any hardware changes to the robot, can be used to improve the user’s telepresence experience, although it introduces certain new challenges. In the following sections, we discuss and elaborate on the results of our experiment.

### Presence

Higher levels of presence and spatial presence were found for the Novel setup when compared to the Standard (Sect. 5.1). These findings differ from previous studies as the setup with a joystick had lower levels of presence than one with a keyboard (Adamides et al. 2017) and varying the user’s reclining angle negatively affected presence due to an increased sensory mismatch because of the use of upright redirection (Luo et al. 2022). Participants believed they felt a greater sense of presence with the Novel setups because of its embodiment and immersion aspects (Sect. 6.1). These findings corroborate with the literature as presence is positively impacted by embodied input methods (Schubert et al. 1999; Hollerbach 2002), where participants ranked rotation method third in importance for presence (Sect. 5.6); and lower lighting conditions can increase immersion (Nordin et al. 2014) and improve presence when the lights are off and volume increased allowing users to be less distracted by their surroundings (Brown and Cairns 2004), where participants ranked lighting and audio as first and second in importance for presence (Sect. 5.6). Conversely, participants mentioned feeling less immersed with the Standard setup because they were *“conditioned”* to associate sitting at a desk and having the lights on to performing a non-immersive task on a computer (Sect. 6.1).

Vection increased across trials and was more intensely sensed when using the Novel setup (Sect. 5.1), which may be due to a number of factors including the reduced inter-sensory cue conflict because of the embodiment (physical rotations) and immersion aspects (darkened room, increased FoV of the displays, more immersive audio device), which participants addressed when discussing the Novel setup (Sect. 6.1). Vection (and potentially motion sickness) can be minimized by providing static backgrounds in the periphery, which serves as a reference frame that the real-world environment is stationary (Prothero and Parker 2003). This may have occurred in the Standard setup as participants mentioned being aware of their surroundings because the lights were on and because speakers were used (Sect. 6.1), lowering their sense of self-motion and sensory conflict (both their peripheral vision of the room and their vestibular sense agreed that they were not moving). The use of two displays in the Novel setup increased the display size and the FoV of the displayed environment. Studies have shown that larger display sizes positively impact presence (Hou et al. 2012) and displays with a larger FoV allow viewers to perceive displayed movement as more physical and exciting (Lombard et al. 1995), actions as more intense (Lombard et al. 1997), and enhance the sensation of vection (Webb and Griffin 2003; Basting et al. 2017). This is relevant because the sensation of vection has a strong correlation to the sensation of presence (Riecke 2010; Hollerbach 2002).

### User experience and usability

#### Felt more motion sickness with the novel setup

Prior research showed that allowing users to physically rotate (as opposed to virtual rotations) not only improves spatial orientation but also helps reduce motion sickness (Rietzler et al. 2018; Ng et al. 2020; Riecke et al. 2010). This motivated us to employ physical rotations for the Novel setup. Unexpectedly, however, results presented higher motion sickness ratings for the Novel setup despite using physical rotations (Sect. 5.2.1), with participants mentioning symptoms of motion sickness such as dizziness and eyestrain (section 6.2.1). Moreover, participants rotated less and slower with the Novel setup (Sect. 5.4), also predicted to reduce motion sickness (Becerra et al. 2020; Kemeny et al. 2017).

Keshavarz et al. (2015) argued that vection is a necessary (but not sufficient) condition for motion sickness to occur. Similarly, increased presence is also known to be associated with increased motion sickness (Keshavarz et al. 2023) as there is a negative relationship between these two measures (Weech et al. 2019). Indeed, participants in the Novel setup found it more immersive and experienced considerably more presence and vection compared to the Standard setup (Sect. 5.1). Conversely, participants in the Standard condition lacked aspects of embodiment (they were stationary) and immersion (lights were on and small FoV of the laptop screen) which might not have been enough to provide a compelling enough sense of vection to allow for motion sickness to occur. Another possible reason for participants feeling motion sick was stated by a participant *“I was rotating a lot with my chair and I was only looking at the monitor and not at the actual world. I was thinking that I was just in the monitor [environment]. I wasn’t dizzy when I was using it, but got dizzy when the lights turned on and it took me back to the real world”* [P42], indicating that it may be influenced by the chair rotation and/or by the quick change from an immersive to a non-immersive environment (from unaware to fully aware of their surroundings). The embodiment aspect of posture/reclining angle may have also affected motion sickness scores between setups, as different reclining angles and the use of upright redirection (where the user’s physical body is angled/not upright but their vision of and movement in the displayed environment is as if they were upright) can affect motion sickness (Luo et al. 2022). There may have also been a mismatch between the participant’s rotation (Sect. 6.2.1) speed and the robot’s rotation. Participants may have rotated faster than the robot, causing a delay in the visuals from matching with their new forward direction as the robot caught up.

Possible strategies to reduce motion sickness can be grouped into several categories. One approach involves deliberately reducing the user’s sense of presence by decreasing the FoV or monitor size (Fernandes and Feiner 2016; Lin et al. 2002; Zhao et al. 2023) or by increasing ambient lighting. A second category focuses on the robot’s control system, such as increasing the maximum rotation speed to better synchronize with user movements (requiring hardware changes) or algorithmically smoothing abrupt changes in translation and rotation (Hashemian et al. 2020, 2024). A third approach involves visualization techniques. These include applying motion blur during rapid movements (Lin et al. 2020; Budhiraja et al. 2017) or using visual anchors—such as static (Luks and Liarokapis 2019; Cao et al. 2018) or dynamic (Cao et al. 2018) rest frames, or a focused area of movement (Park et al. 2022) — to decrease optical flow. However, these latter visual techniques carry a notable trade-off, as they may negatively impact immersion, presence, and the overall user experience (Rouhani et al. 2024).

Future research is needed to test these hypotheses and compare them with setups of various levels of immersion. For example, rather than simply having the lights on or off, we could use different brightness levels. We speculate that increasing brightness would decrease immersion and, consequently, reduce motion sickness. We could also use displays of different sizes and levels of immersion, such as a VR HMD or a large curved/panoramic display. We speculate these would increase presence and motion sickness as the FoV and the mismatch between user rotation input and robot rotation increase.

Although results showed higher levels of motion sickness for the Novel setup, the overall motion sickness values (total SSQ) were low.

#### Novel setup was more physically demanding

The Standard setup was found to be less tiring, with muscles more relaxed (Sect. 5.2), and presented lower task load ratings than when using the Novel setup (Sect. 5.3.1). Some participants believed this was due to the Novel setup having more physically demanding embodiment aspects, for instance, moving their head to look between screens, using their wrist to push and pull the joystick, and their legs to turn the chair (Sect. 6.2.2). Conversely, participants felt the Standard setup had less physically demanding embodiment aspects, requiring only simple eye-movements to look between camera views and their fingers to press keys on a keyboard. This may also be due to the cognitive and physical resources required by the setup and its level of presence, as our results corroborate previous studies where the number of attentional sources (i.e., visual, auditory, and mental), the amount of effort/energy required to perform tasks (Brown and Cairns 2004), and the willingness of users to use their mental resources (Green and Jenkins 2020) correlate with the level of presence users feel, as all these ratings were higher for the Novel setup.

#### Novel setup is intuitive and precise

Participants found the Novel setup’s embodiment aspect of physically rotating to control rotation and immersion aspect of using two screens in different positions to view the displayed environment as intuitive as they both mimicked how people naturally rotate and look around in real life, such that some participants said that the Novel setup felt as if they were using VR without an HMD (Sect. 6.2.6). Participants felt more control and precision with the embodiment aspect of the Novel setup’s input methods, as they could move at a pace of their choosing, particularly helpful when making small movements and avoiding obstacles. Though the Standard setup was rated by participants as easier to control, the Novel setup was rated (Sect. 5.3), described by participants (Sect. 6.2.6), and shown to be more precise in the performance measures with less collisions and accumulated rotations (Sect. 5.4), which may be due to the embodiment aspects of its input devices. The Standard setup’s keyboard input, which participants mentioned require minimal effort from their fingers, offered digital signals (discrete values of either 0 or maximum speed and not a continuous range) based on a key being pressed or not. This matches what participants discussed as they found the Standard setups’ inputs were always at maximum speed, making it difficult, imprecise, and causing overshoots that required corrections. The Novel setup’s joystick and IMU (chair rotation), which participants stated required more effort from their legs (especially to reach maximum speeds), provided analog signals (a continuous range of values from 0 to maximum speed) based on the joystick’s pitch angle to move the robot forward/backward or the IMU’s yaw angle to rotate the robot. The amount of effort required from these input devices may be the reason why the Standard setup was rated as being easier to control.

#### Novel setup is more engaging, enjoyable, and exciting

Participants found the Novel setup to be more engaging, enjoyable, and exciting (Sect. 5.2), even though motion sickness, which participants felt more of with this setup (Sect. 5.2.1), can negatively affect user experience (Lee et al. 2024). This may be because participants felt more present, immersed, and engaged when using the Novel setup, as studies have shown a connection between these sensations with enjoyment (Wirth et al. 2007; Merikivi et al. 2017; Heeter 1992), which corroborates with our findings of the Novel better supporting presence and immersion. Conversely, participants found the Standard setup to be boring, comparing it to a chore they had to perform, while the Novel setup felt new and exciting, especially due to the embodiment aspect of physically moving to rotate (Sects. 6.2.7 and 6.1). Participants may have also enjoyed the Novel setup more because of its ease of use (Merikivi et al. 2017), as participants found the Novel setup intuitive and easy to learn (Sect. 6.2.6), while finding the Standard more confusing, complicated, and less precise (Sect. 5.3).

It is important to consider that a possible reason for these favorable results towards the Novel setup may be due to the Standard setup being composed of factors familiar to participants (Sect. 6.2.6) and they are *“conditioned”* to using a computer in this manner when performing a non-immersive task (Sect. 6.1). There may also be a "Novelty Effect", where a positive effect occurs because of the newness of the innovation rather than the innovation itself (Elston 2021). This could be due to participants being exposed to specific new factors, such as using two vertically placed displays to view a simulated environment from different perspectives or using physical rotation as an input method, or experiencing all of the Novel setup factors at once. Studies have shown that novelty can impact motivation (Fierro-Suero et al. 2020), enjoyment (Li et al. 2021), interest (Sung et al. 2019), and satisfaction (Fierro-Suero et al. 2020).

#### Novel setup is less safe, comfortable, and would not be used regularly or for longer periods

The Novel setup was rated as less safe (Sect. 5.3), which participants felt was because of the immersion aspect of having the lights off (Sect. 6.2.5), making them anxious of their surroundings (not knowing who/what is around them) and if they would hit something while rotating in the chair (a common safety concern participants have in VR studies (Huang et al. 2018; Scavarelli and Teather 2017; Jelonek 2023). Future studies could investigate how varying the level of lighting (how much of the surrounding environment the user can see) can impact the user’s sense of immersion, presence, and safety.

Research has shown that using a chair in a reclined position and having the display at eye-level increases comfort (Bendix et al. 1985; Haynes and Williams 2008; Kothiyal and Bjørnerem 2009; Imamov et al. 2020), which led us to include similar features in our Novel setup. Participants discussed various embodiment and immersion aspects of the Novel setup that were comfortable (Sect. 6.2.4), such as their reclined and relaxed posture, being able to see the displayed environment well without having to lean into the screen (as some had to do with the Standard setup), being able to freely move, and feeling more in control due to precise input. Although more participants described the Novel setup as more comfortable than the Standard in the interview, they rated the Standard setup as more comfortable in the survey (Sect. 5.3). This difference may have been because the Novel setup was more physically demanding for participants and caused more motion sickness (Sects. 5.3.1 and 5.2.1), especially over time, which were concerns participants expressed, to the extent that these negative aspects outweighed the positive ones, making the setup less comfortable overall.

The Novel setup was also rated lower for regular and long term use (Sect. 5.3), which participants believed would be due to feeling too tired, from the physical demand (from using their legs to rotate), and motion sick, especially with more/longer session times (Sect. 6.2.3). Moreover, participants felt that using the Standard setup for long periods of time would be uncomfortable due to the strain on their fingers from all the input and from staying in the same posture (embodiment aspects). Some participants mentioned they could use the Novel setup for longer periods as it was more immersive and entertaining, but the duration would depend on how physically demanding the task would be.

### Performance

#### Collisions, distance traveled, accumulated rotations

Participants collided, traveled, and rotated less with the Novel setup, by the second trial, and had a higher average linear and rotation speed with the Standard setup, throughout all trials (Sect. 5.4). These results differ from the literature where keyboard setups outperformed joysticks (Thrash et al. 2015; Ruddle and Lessels 2009) and the difference in reclining angle and use of upright redirection has shown to negatively affect spatial perception of distance, direction, and position (Luo et al. 2022). This mismatch with the literature may be due the setups’ embodiment aspects of input methods and laptop location, and the immersion aspect of the number of displays.

The setups’ translation and rotation input methods, ranked second and first in importance for performance (Sect. 5.6), may have affected their average linear and rotation speeds, consequently impacting the number of collisions. The digital signal of the Standard setup’s keyboard may have resulted in higher average linear and rotation speeds, as participants mentioned less precise control of the robot’s movements with it compared to the precise control with the Novel setup’s input methods (Sects. 5.3 and 6.2.6). This may have caused participants using the Standard setup to collide and rotate more, especially in narrow locations. Because physical rotations improves spatial updating (Chance et al. 1998; Riecke et al. 2010), proprioceptive cues, and distance judgment (Hollerbach 2002), this may have better supported participant’s spatial presence when using the Novel setup (Sect. 5.1), requiring them to look around less to understand their environment, leading to less rotations and collisions. This coincides with participants feeling their body movements, specifically rotation, were natural and precise, which provided a stronger sense of presence and made obstacle avoidance easier (Sects. 6.1 and 6.2.6). This aligns with previous literature where telepresence robot users with a sense of presence better spatially understand and maneuver around a remote environment (Nostadt et al. 2020). Participants also mentioned looking often at the down-facing camera view to avoid obstacles because they weren’t familiar with the robot’s body size, which they do not need to do when walking in the real world (Sects. 6.3).

The number of displays, ranked third in importance for performance (Sect. 5.6), may have led to the Novel setup colliding less, as participants mentioned that using two displays (given their larger size, wider FoV, and position) improved their performance as it was easier to see things (Sect. 6.3). Participants felt the Standard setup’s screens were too condensed and confusing (Sect. 6.2.6), which may have led to more collisions. These findings may be explained by previous studies where displays with larger FoVs were shown to improve target detection and identification performance (Ragan et al. 2015), reduce the number of collisions (Nagahara et al. 2003), require less navigation commands (Bazzano et al. 2019), and enhance distance judgment (Masnadi et al. 2022). Larger displays also increase attention, in particular selective attention to content in the viewer’s peripheral vision, as it is more responsive to changes than the foveal vision (Livingstone and Hubel 1988), which coincides with a participant’s use and description of the Novel setup’s bottom screen as peripheral vision and may have helped with obstacle avoidance.

#### Task completion time

Considering all three trials together, participants completed tasks faster with the Standard setup (Sect. 5.4). This finding is consistent with previous studies in which keyboard locomotion outperformed a joystick (Thrash et al. 2015; Adamides et al. 2017). However, this initial advantage was not static. While participants improved with both setups over time, the performance gap narrowed significantly. By the third trial, task completion times for the Novel and Standard setups had converged to statistically similar levels. This rapid improvement in the Novel setup might be attributed to participants colliding less frequently and for shorter periods of time, traveling less, rotating less, and increasing their average linear speed over time as they practiced. The results of the third trial align with those of studies comparing virtual translation and rotation (similar to the Standard setup) to virtual translation and physical rotation (similar to the Novel setup) (Riecke et al. 2010).

#### Performance and energy indexes

By the second trial, the Novel setup had shown benefits over the Standard in terms of performance and energy efficiency indexes, and these advantages increased further in the third trial. The Novel setup may have performed better due to cognitive demand and learning effects, as participants may have become familiar enough with it to focus more on their task than on operating the robot (Cohen et al. 2011). This would allow them to allocate more cognitive resources to their task, resulting in improved performance. Regarding energy efficiency, in theory, participants would use less energy with the Novel setup, meaning they would have more battery power available for longer use of the robot and/or lower operating costs compared to the Standard setup.

The Novel setup required approximately nine minutes of use — the duration of three trials — for its performance to match that of the Standard setup. This brief learning period suggests that operators could likely achieve proficiency within a single, typical telepresence session, which often exceeds 20 min (Jones et al. 2020; Yang et al. 2018; Shi et al. 2019; Cash and Prescott 2019). However, this result should be treated with caution as further research is needed to investigate the effect of additional training time on performance and to establish whether longer sessions would be feasible when considering factors such as physical demand and motion sickness. Future studies could also investigate whether the Novel setup continues to reduce cognitive demands and improve performance to the extent that it surpasses task and collision times compared with the Standard setup. If so, how long would it take to reach this point and when would the learning effects, and consequently the improved performance measures, saturate and plateau?

### The effects of embodiment and immersion setup factors

Our results show that the setup factors related to embodiment and immersion, at least when put together, contribute towards a better telepresence robot experience. However, certain metrics were worse with the Novel setup and we only tested the system as a whole. The rankings provide initial ideas of each individual factor’s level of contribution, but further studies are needed, where one factor is varied at a time, to better assess their individual impact and their importance when designing the user interface.

As an implication of the results of this study, one could ask the following practical question: Is it worth using the Novel setup, which provides a better sense of presence, user experience, and performance than a Standard setup, but is less comfortable and has higher motion sickness ratings, task load demands, and needs some brief initial training? In the context of this study, we can answer that it depends on the user’s purpose for operating a telepresence robot and the session duration. While our study’s task was performance-focused, there was no statistically significantly preference between the setups for this type of use. Considering duration, participants indicated they would use the Standard setup more regularly and for longer periods of time because they would probably get too motion-sick or tired with the Novel setup (6.2.3). Although our task was not focused on social interactions, when asked, some participants thought they would prefer to use the Standard setup because they would have better control of the robot and could rotate faster to talk to people behind them, while some others would prefer the Novel setup because they want to feel present and focused when talking to people and not *"like a zoom video"* with the Standard setup (Sect. 6.4.1). Further studies focused on social scenarios are needed to better access participants’ setup preference as our findings are based on what some participants assume they would prefer in imagined scenarios.

Regarding the Novel setup’s hardware components required for its use, although the hardware components required for the Novel setup may seem like a possible adoption barrier for non-technical users or organizations without technical support at first, we believe it is not. The secondary screen, IMU, and joystick used were "plug and play", meaning they could be used just by connecting them as the software managed the integration and all the user would need to do is place things at a comfortable position. To further facilitate this setup and user experience, a simpler version of this setup is also possible. For example, the headphones and secondary display could be what the user has available in their home/office (i.e., a phone or tablet). Rather than using an external IMU to measure rotation, it could be obtained from the secondary display’s internal IMU. Instead of using the platform on the chair’s armrest to position the laptop and joystick, a laptop stand placed on the user’s lap and a smaller, lighter joystick on the armrest or a large tray on the user’s lap could be used. A relatively powerful laptop and a cabling system, to constantly power the laptop, was required because the simulation was resource-intensive. However, this would not be necessary for real-world use because there would be no simulation and, therefore, the laptop’s battery would suffice, eliminating the need for a power cable and preventing it from tangling while the user rotates.

### Limitations

As with all evaluations, this study has its limitations and we want to identify the most important ones that we are aware of as they may affect the generalization of our findings. First, due to the simplicity of our task (i.e., no dynamic objects or complex winding and narrowing paths), it did not require much backward or rapidly changing movements, and the task was fairly predictable and did not have a high degree of difficulty. Second, as previously discussed, because this study focused on two different setups, the Standard and the Novel setup, which are composed of seven different factors, the results need to be interpreted regarding these setups as a whole and not on their individual factors. Third, as the purpose of the audio was to help immerse the participants, rather than being required for the performance of tasks such as perceiving an alarm and finding its source, or using audio cues to better perceive collisions, this may explain why the audio was rated as the least important for performance. Fourth, future research is needed to investigate to what degree our findings might generalize to different types of robots (i.e., treaded, bipedal humanoids, or robots with multiple limbs for navigation), as the mismatch between the user’s chair rotation and the robot’s rotation/motion may lead to operational challenges, failure, and/or motion sickness. Finally, conducting the study in a simulated environment can be considered a limitation because the Novel setup has not yet been tested with a real robot. However, the modeling of the simulated robot closely followed that of existing telepresence robots, and the simulated environment enabled greater control over confounding variables and the environment. Future work will involve testing the Novel setup with a real robot to examine the impact of factors such as vibration, hardware noise, terrain variation (e.g. smoothness and unevenness), and uncertain motion (e.g. slippage). We anticipate that latency- and terrain-related issues may cause a mismatch between the user’s physical pose and what they see in the remote environment, which could lead to motion sickness. Although hardware noise may annoy users, it could positively impact their sense of presence, as sounds from the remote environment may enhance their sense of being there. While we anticipate differences between the simulation and real-world use, these should not significantly impact our results, as they would affect both the Novel and Standard setups similarly.

## Conclusion

This study investigated how the interface used to operate a telepresence robot affects the user’s overall experience across multiple measures, including presence, user experience, usability, and performance. Two different setups were used in this study, a Standard setup consisting of factors commonly found in commercial telepresence robots, and a Novel setup, whose factors incorporate insights from VR and robotics. Results showed that by the end of the third trial, the Novel setup significantly improved the user experience, the user’s sense of presence and self-motion, and performance compared to the Standard setup, but it also caused participants to experience more motion sickness, though generally low values, and task load demands. After only about nine minutes of practice (by the third trial), participants performed better overall when using the Novel setup. However, the Standard setup was found to be a setup that could be used more regularly and for longer periods of time, but was less precise and more complicated and confusing to use. Although our study did not focus on social situations, participants believed they would prefer to use the Novel setup for social uses. We plan to investigate social interactions in a future study, which we believe will more effectively showcase the strength of the Novel in terms of embodiment and immersion. Because the setups were considered as a whole, the results of this study provides a basis for researchers to further investigate the impact of each setup factor. This also provides a basis for designers/roboticists to create robot user interfaces focusing on the user’s overall experience. This study also showed how existing telepresence robot user interfaces can be improved with simple methods and low-cost tools that do not require changes to the robot’s hardware, just changes to the user’s setup.

While this study employed a home-inspection scenario, its findings address the universal challenge of remote navigation and are therefore relevant to any domain where effective teleoperation is critical—including industrial inspection, education, healthcare, and remote collaboration. More broadly, our research provides a key insight for the fields of human-computer and human-robot interaction. It demonstrates that a holistic design approach, prioritizing operator embodiment and immersion through low-cost, accessible modifications, can fundamentally improve the telepresence experience. This user-centric paradigm offers a path toward designing more effective and engaging human-robot interfaces that move beyond the limitations of "Skype on wheels”.

## Supplementary Information

Below is the link to the electronic supplementary material.

Supplementary file 1 (pdf 183 KB)

Supplementary file 2 (mp4 32859 KB)

## Data Availability

No datasets were generated or analysed during the current study.
