# Supplementary material for: From “skype on wheels” to embodied telepresence: a holistic approach to improving the user experience of telepresence robots
Source: Virtual Real. 2025 Sep 25;29(4):161. doi: 10.1007/s10055-025-01222-0 (PMC12464061; doi:10.1007/s10055-025-01222-0)
Supplement: Supplementary file 1 — (pdf 183 KB) [file 10055_2025_1222_MOESM1_ESM.pdf]

Supplementary Material A: User Study Dependent Variables

Title: From "Skype on Wheels" to Embodied Telepresence: A Holistic Approach to Improving the User Experience of Telepresence Robots

Journal: Virtual Reality

Authors: Ivan A. Aguilar, Markku Suomalainen, Steven M. LaValle, Timo Ojala, Bernhard E. Riecke

Corresponding Author: Ivan A. Aguilar (iaguilar@sfu.ca), Simon Fraser University, British Columbia, Canada

1 Dependent Variables

The following tables provide information on what group the variable is a part of, how it was measured/-calculated/asked (research instrument), and what questionnaire or study it is based on. The variable groups are: presence, user experience (shown together in Table 1), usability (Table 2), and setup factor importance and performance (shown together in Table 3).

Table 1 Presence and User Experience measures on how they were measured and what questionnaire/study they are based on.

Group	Measure	Research Instrument	Based On
**Presence**	Presence	"I was no longer aware of my real environment"	Adhikari et al. (2022)
	Presence (Overall Presence)	All IPQ Questions	Schubert et al. (2001)
	Spatial Presence	Spatial Presence IPQ Questions	Schubert et al. (2001)
	Presence	"I felt more present in the simulated environment with which setup?"	Created for this study
	Vection Intensity	"I had a strong sensation of self-motion with the setup. (It felt like I was moving across the simulated environment rather than the simulated environment was moving towards me.)"	Riecke and Feuereissen (2012)
**User Experience**	Tiredness	"I felt tired after using the setup"	Adhikari et al. (2022)
	Relaxed Muscles	"My muscles were relaxed"	
	Comfort	"The setup was comfortable to use"	Bowman et al. (1999)
	Engagement	"The setup helped me to be involved and engaged"	Adhikari et al. (2022)
	Enjoyment	"I enjoyed using the setup"	Laugwitz et al. (2008)
	Excitement	"How would you rate this setup on how exciting it was to use?"	Laugwitz et al. (2008)
	Motion Sickness	Simulator Sickness Questionnaire (SSQ) - Total and Subscales (Disorientation, Nausea, Oculomotor)	Kennedy et al. (1993)

Table 2 Usability and Use Case Setup Suitability measures on how they were measured and what questionnaire/study they are based on.

Group	Measure	Research Instrument	Based On
Usability	Safety	"The setup was safe to use"	
	Regular Use	"I think I would like to use the setup regularly"	Brooke et al. (1996); Adhikari et al. (2022)
	Long Term Use	"I could imagine using the setup for longer time periods"	Adhikari et al. (2022)
	Complicated	"How would you rate this setup on how complicated it was to use?"	Laugwitz et al. (2008)
	Confusing to use	"How would you rate this setup on how confusing it was to use?"	Laugwitz et al. (2008)
	Precise Control	"I had precise control of my movements"	Hashemian et al. (2023)
	Overall Usability	"The overall usability of the setup was high"	Hashemian et al. (2023)
	Movement Speed	"What did you think of the robot's movement speed?"	Chin et al. (1988)
	Easy Obstacle Avoidance	"It was easy to move across the simulated environment without hitting anything"	Brooke et al. (1996)
	Task Efficiency	"How would you rate this setup on how efficient it was in allowing you to complete the task given in this study?"	Laugwitz et al. (2008)
	Task Support	"How would you rate this setup on how supportive it was in allowing you to complete the task given in this study?"	Laugwitz et al. (2008)
	Ease of Learning	"It was intuitive/easy to learn how to navigate with the setup"	Chin et al. (1988)
	Easy Task Concentration	"I could easily concentrate on the task"	Adhikari et al. (2022)
	Task Load	NASA-Task Load Index Questionnaire - Overall and individual measures (Mental, Physical, and Temporal, and Performance, Effort and Frustration)	Hart and Staveland (1988)
	Easier to Control	"It was easier to drive/control the robot with which setup?"	Created for this study
Use Case Setup Suitability	Travel or Sightseeing	"Telepresence travel/sightseeing (remotely exploring places you would like to visit)"	Created for this study
	Personal Social Situations	"Personal social situations (meeting with friends and family)"	
	Small Professional Gatherings	"Professional small gatherings (business visit in a different city)"	
	Large Professional Gatherings	"Professional large gatherings (conferences/events)"	
	Task-focused	"Completing tasks like the one in this study (navigate across a home, find objects, and avoid hitting obstacles)"	

Table 3 Performance (raw), Efficiency Indexes (processed), and Setup Factor importance measures on how they were measured and what questionnaire/study they are based on.

Group	Measure	Research Instrument	Based On
**Performance**	Task Completion Time	How long it took to complete the trial (not including time spent in the UI answering questions)	Bowman et al. (1999); Nowak et al. (2010)
	Number of Collisions	How many times collided with objects	Desai et al. (2011); Nowak et al. (2010)
	Collision Time	Total time (seconds) spent ”in collisions” (in direct contact with an object other than the floor)	Bailey and Witmer (1994)
	Distance Traveled	Total distance traveled (meters)	Nguyen-Vo et al. (2019); Nowak et al. (2010)
	Average Linear Speed	Average linear speed (meters/second), not including time spent in the UI answering questions	Nguyen-Vo et al. (2019)
	Accumulated Rotations	Total yaw rotations (euler angles)	Nguyen-Vo et al. (2019)
	Average Rotation Speed	Average rotation speed (yaw euler angle/second), not including time spent in the UI answering questions	Created for this study
**Efficiency Indexes**	Task Completion Time	Ratio of Standard setup's by Novel setup's completion time	Created for this study
	Number of Collisions	Ratio of Standard setup's by Novel setup's number of collisions	
	Distance Traveled	Ratio of Standard setup's by Novel setup's distance traveled	
	Accumulated Rotations	Ratio of Standard setup's by Novel setup's accumulated rotations	
	Performance	Geometric mean of the task completion time and number of collisions efficiency indexes	
	Energy	Geometric mean of the task completion time, number of collisions, distance traveled, and accumulated rotations efficiency indexes	
**Setup Factor Importance**	Presence	”How important do you think each of the following characteristics are for supporting a sense of being in the simulated environment?”	Created for this study
	Performance	”How important do you think each of the following characteristics are for performing the task you were given in this study (navigate across a home, find objects, and avoid hitting obstacles)?”	

2 Efficiency Index

The efficiency indexes were calculated by normalizing performance measures (using a ratio of the Standard's performance divided by the Novel's), where a value greater or less than one indicates that either the Novel or Standard setup was more efficient for that trial, respectively. Based on these efficiency indexes we created to measures, the performance and the energy efficiency indexes. Calculated via Equation 1,

$$\text{Performance Efficiency Index} = \sqrt{\frac{T_{Standard}}{T_{Novel}} \times \frac{(C_{Standard}+1)}{(C_{Novel}+1)}} \quad (1)$$

$$\text{Energy Efficiency Index} = \sqrt[4]{\frac{T_{Standard}}{T_{Novel}} \times \frac{(C_{Standard}+1)}{(C_{Novel}+1)} \times \frac{D_{Standard}}{D_{Novel}} \times \frac{R_{Standard}}{R_{Novel}}} \quad (2)$$

where,
T = task completion time,
C = number of collisions,
D = distance traveled,
R = accumulated rotation.

the performance efficiency index combines two measures: task completion time and the number of collisions. These measures were chosen as participants were instructed to complete tasks as quickly as possible while minimizing collisions. Calculated via Equation 2, the energy efficiency index combines 4 measures: task completion time, number of collisions, distance traveled, and accumulated rotation. These measures were chosen as they would each impact how much energy the robot would consume during operation.

The multiplication of the efficiencies and its result by the nth root (based on the number of measures considered in the equation), known as the geometric mean, provides the final efficiency index value. To handle zeros while maintaining ratio integrity in the collision calculation, we used a "plus-one smoothing" method where there is an addition of 1 to both setup values before the ratio is calculated.

References

Adhikari, A., Zielasko, D., Aguilar, I., Bretin, A., Kruijff, E., Heyde, M., Riecke, B.E.: Integrating continuous and teleporting vr locomotion into a seamless 'hyperjump'paradigm. IEEE Transactions on Visualization and Computer Graphics **29**(12), 5265–5281 (2022)

Brooke, J., *et al.*: Sus-a quick and dirty usability scale. Usability evaluation in industry **189**(194), 4–7 (1996)

Bowman, D.A., Davis, E.T., Hodges, L.F., Badre, A.N.: Maintaining spatial orientation during travel in an immersive virtual environment. Presence **8**(6), 618–631 (1999)

Bailey, J.H., Witmer, B.G.: Learning and transfer of spatial knowledge in a virtual environment. In: Proceedings of the Human Factors and Ergonomics Society Annual Meeting, vol. 38, pp. 1158–1162 (1994). SAGE Publications Sage CA: Los Angeles, CA

Chin, J.P., Diehl, V.A., Norman, K.L.: Development of an instrument measuring user satisfaction of the human-computer interface. In: Proceedings of the SIGCHI Conference on Human Factors in Computing Systems, pp. 213–218 (1988)

Desai, M., Tsui, K.M., Yanco, H.A., Uhlik, C.: Essential features of telepresence robots. In: 2011 IEEE Conference on Technologies for Practical Robot Applications, pp. 15–20 (2011). https://doi.org/10.1109/TEPRA.2011.5753474

Hashemian, A.M., Adhikari, A., Kruijff, E., Heyde, M.v.d., Riecke, B.E.: Leaning-based interfaces improve ground-based vr locomotion in reach-the-target, follow-the-path, and racing tasks. IEEE Transactions on Visualization and Computer Graphics **29**(3), 1748–1768 (2023) https://doi.org/10.1109/TVCG.2021.3131422

Hart, S.G., Staveland, L.E.: Development of nasa-tlx (task load index): Results of empirical and theoretical research. In: Hancock, P.A., Meshkati, N. (eds.) Human Mental Workload. Advances in Psychology, vol. 52, pp. 139–183. North-Holland, ??? (1988). https://doi.org/10.1016/S0166-4115(08)62386-9

Kennedy, R.S., Lane, N.E., Berbaum, K.S., Lilienthal, M.G.: Simulator sickness questionnaire: An enhanced method for quantifying simulator sickness. The international journal of aviation psychology **3**(3), 203–220 (1993)

Laugwitz, B., Held, T., Schrepp, M.: Construction and evaluation of a user experience questionnaire. In: Symposium of the Austrian HCI and Usability Engineering Group, pp. 63–76 (2008). Springer

Nguyen-Vo, T., Riecke, B.E., Stuerzlinger, W., Pham, D.M., Kruijff, E.: Naviboard and navichair: Limited translation combined with full rotation for efficient virtual locomotion. IEEE transactions on visualization and computer graphics **27**(1), 165–177 (2019)

Nowak, W., Zakharov, A., Blumenthal, S., Prassler, E.: Benchmarks for mobile manipulation and robust obstacle avoidance and navigation. BRICs Deliverable D **3**, 1 (2010)

Riecke, B.E., Feuereissen, D.: To move or not to move: can active control and user-driven motion cueing enhance self-motion perception (" vection") in virtual reality? In: Proceedings of the ACM Symposium on Applied Perception, pp. 17–24 (2012)

Schubert, T., Friedmann, F., Regenbrecht, H.: The experience of presence: Factor analytic insights. Presence: Teleoperators & Virtual Environments **10**(3), 266–281 (2001)
